# Superacid treatment of dielectric-encapsulated monolayer molybdenum disulfide for enhanced photoluminescence

**DOI:** 10.1039/d6tc02199a

**Published:** 2026-09-25

**Authors:** Brendan F. M. Healy, Kelsey J. Hall, Sophie L. Pain, Nicholas E. Grant, John D. Murphy

**Affiliations:** a School of Engineering, University of Warwick Coventry CV4 7AL UK brendan.f.m.healy@warwick.ac.uk; b School of Engineering, University of Birmingham, Edgbaston Birmingham B15 2TT UK

## Abstract

With a direct bandgap and strong photoluminescence (PL) emission, monolayer molybdenum disulfide (1L MoS_2_) is a promising material for next-generation optoelectronic technologies. However, the intrinsically low PL quantum yield (PLQY) and the requirement for dielectric integration present significant challenges in the realisation of practical MoS_2_-based devices. Chemical treatment with the superacid bis(trifluoromethanesulfonyl)amide (TFSA) is known to enhance dramatically the PL emission of 1L MoS_2_ through defect passivation and p-type doping. Conversely, atomic layer deposition (ALD) of a dielectric material can introduce defect states and induce n-type doping, thus degrading the MoS_2_ optical performance. Despite the competing effects of these two processing routes, the combined impact of superacid treatment and ALD dielectric growth on 1L MoS_2_ has not been thoroughly studied. In this work, we reveal the instability of the TFSA-enhanced MoS_2_ PL intensity during subsequent ALD of aluminium oxide (Al_2_O_3_), due to thermal exposure and precursor interaction. In contrast, we demonstrate that TFSA treatment of thermal Al_2_O_3_-encapsulated 1L MoS_2_ can successfully enhance the PL intensity by up to two orders of magnitude, even for dielectric films of up to ∼96% surface coverage. This enhancement is suppressed only when the Al_2_O_3_ layer becomes sufficiently thick (>∼40 nm) and approaches complete coalescence, preventing access of TFSA-derived species to the underlying MoS_2_. This work establishes an effective encapsulation/passivation strategy for enhancing the optical performance of dielectric-covered 1L MoS_2_, offering a practical pathway for integrating chemical passivation into device-relevant architectures.

## Abstract 2

Post-deposition superacid treatment restores and strongly enhances the photoluminescence of dielectric-encapsulated monolayer MoS_2_, enabling effective chemical passivation in device-relevant structures.

## Graphical abstract

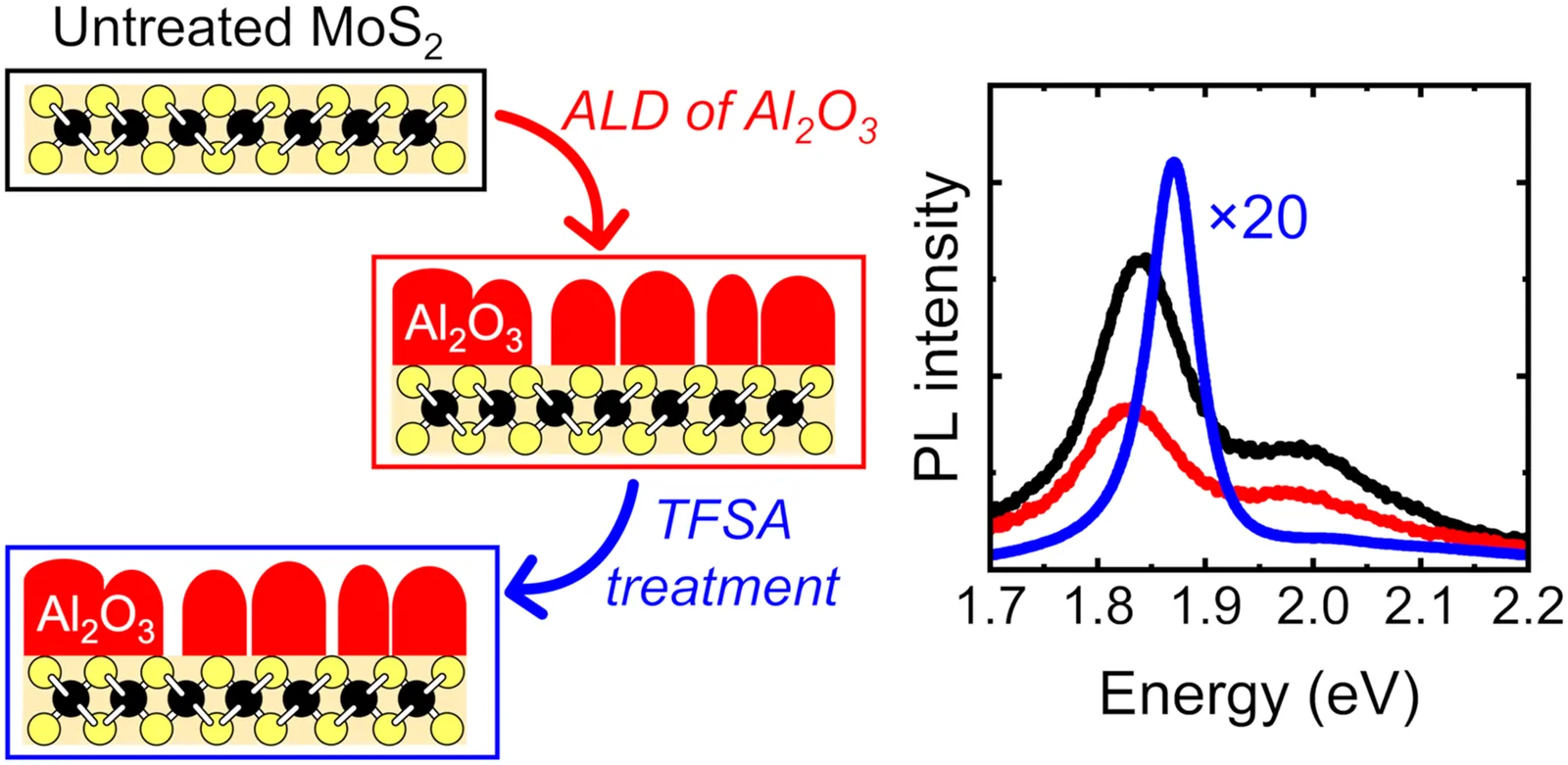

## 1. Introduction

Exhibiting novel optical and electrical properties at single-layer thickness, semiconducting transition metal dichalcogenides (TMDCs) have attracted significant research interest for integration in future optoelectronic devices. Among them, monolayer molybdenum disulfide (1L MoS_2_) is particularly promising due to its direct bandgap of ∼1.8 eV and resulting photoluminescence (PL) emission,^1,2^ enabling applications in light-emitting devices,^3,4^ photovoltaics,^5–8^ photodetectors,^9–11^ and phototransistors.^12–14^ Scalable device production requires large-area TMDC growth, and 1L MoS_2_ is routinely synthesised *via* chemical vapour deposition (CVD). Compared to mechanically exfoliated monolayers, CVD-grown 1L MoS_2_ possesses a high defect density,^15,16^ predominantly sulphur (S) vacancies.^17^ Such defects trap photoexcited carriers and facilitate non-radiative recombination, with the PL quantum yield (PLQY) from 1L MoS_2_ reported to be as poor as <1%.^1,18^ While modifications to the growth conditions can offer modest improvements in the PL emission efficiency from CVD-grown 1L MoS_2_,^19^ substantial enhancement of the PLQY is required for efficient, high-performing 1L MoS_2_-based optoelectronic devices.^20,21^

Treatment with the organic superacid bis(trifluoromethanesulfonyl)amide (TFSA) is widely recognised as an effective chemical passivation strategy for a range of electronic materials,^22^ including monolayer TMDCs.^17,23–25^ Immersion in TFSA solution has been shown to yield ≥95% PLQY in 1L MoS_2_,^26^ enhancing the PL intensity by up to two orders of magnitude.^17,23–25^ We recently revealed the enhanced PL behaviour exhibits long-term stability under ambient conditions.^27^ TFSA can also significantly improve MoS_2_-based device performance, with a five-fold increase in electron mobility reported after superacid treatment.^28^

Many practical optoelectronic devices, particularly those based on the field-effect transistor (FET), require integration of a gate dielectric.^29^ High dielectric constant (high-*κ*) materials such as hafnium oxide (HfO_2_), aluminium oxide (Al_2_O_3_), and zinc oxide (ZnO) are commonly grown *via* atomic layer deposition (ALD). Traditional thermal ALD of high-*κ* dielectrics on monolayer TMDCs is notoriously challenging. The chemically inert basal plane of 1L MoS_2_ lacks the dangling bonds required for initial precursor chemisorption.^30,31^ ALD of dielectrics directly on 1L MoS_2_ is therefore dominated by precursor physisorption and proceeds *via* an island growth mode, yielding incomplete layers even at high cycle numbers.^31,32^ Ultraviolet-ozone (UV-O_3_) pre-treatment,^33–35^ plasma-enhanced ALD (PEALD),^36,37^ and low-temperature ALD^30,38^ can improve nucleation but may compromise MoS_2_ material quality and degrade the dielectric/MoS_2_ interface. Reliable ALD integration of high-*κ* dielectrics remains an important challenge for realisation of practical 1L MoS_2_-based optoelectronic devices.

Harnessing the enhanced optoelectronic performance of superacid-treated 1L MoS_2_ in a practical device architecture may necessitate combining dielectric deposition with TFSA exposure. We previously revealed that superacid treatment and ALD-dielectrics separately induce opposing changes to the CVD-1L MoS_2_ PL emission, arising from differing charge doping effects.^23^ TFSA treatment enhances, blueshifts, and sharpens the MoS_2_ PL emission, consistent with depletion of the electron density – a p-type doping effect. In contrast, thermal ALD of Al_2_O_3_ or HfO_2_ reduces, redshifts, and broadens the PL signal, indicating n-type doping. Therefore, it is important to ascertain the overall effect on the optical behaviour of 1L MoS_2_ following both ALD-dielectric growth and superacid treatment.

The processing order must be considered when combining superacid and ALD processing of 1L MoS_2_. ALD of a dielectric on TFSA-enhanced 1L MoS_2_ may degrade the improved PL behaviour *via* vacuum exposure,^39^ elevated temperature, and/or reactive precursors. Alharbi *et al.* demonstrated ALD of HfO_2_ on TFSA-treated MoS_2_ in the fabrication of top-gated FETs,^40^ citing previous indications that TFSA treatment exhibits thermal stability up to 300 °C. While improved electrical performance was observed, no optical characterisation was reported to verify whether the TFSA-enhanced PL emission survived the ALD process. As such, it remains crucial to determine if the elevated PL character of TFSA-treated 1L MoS_2_ can survive the processing conditions of subsequent dielectric growth. Reversal of the processing sequence – treating dielectric-capped 1L MoS_2_ with TFSA – may offer a pathway to enhanced 1L MoS_2_ optical performance in a device-relevant architecture. While TFSA treatment of uncovered and polymer-capped 1L MoS_2_ is well-described in the literature,^25,41,42^ the impact on dielectric-encapsulated 1L MoS_2_ remains poorly established. Comprehensively evaluating how dielectric morphology, thickness, and coverage influence the optical characteristics of 1L MoS_2_ after subsequent TFSA treatment is key for translating chemical passivation strategies into practical devices.

**Fig. 1:**
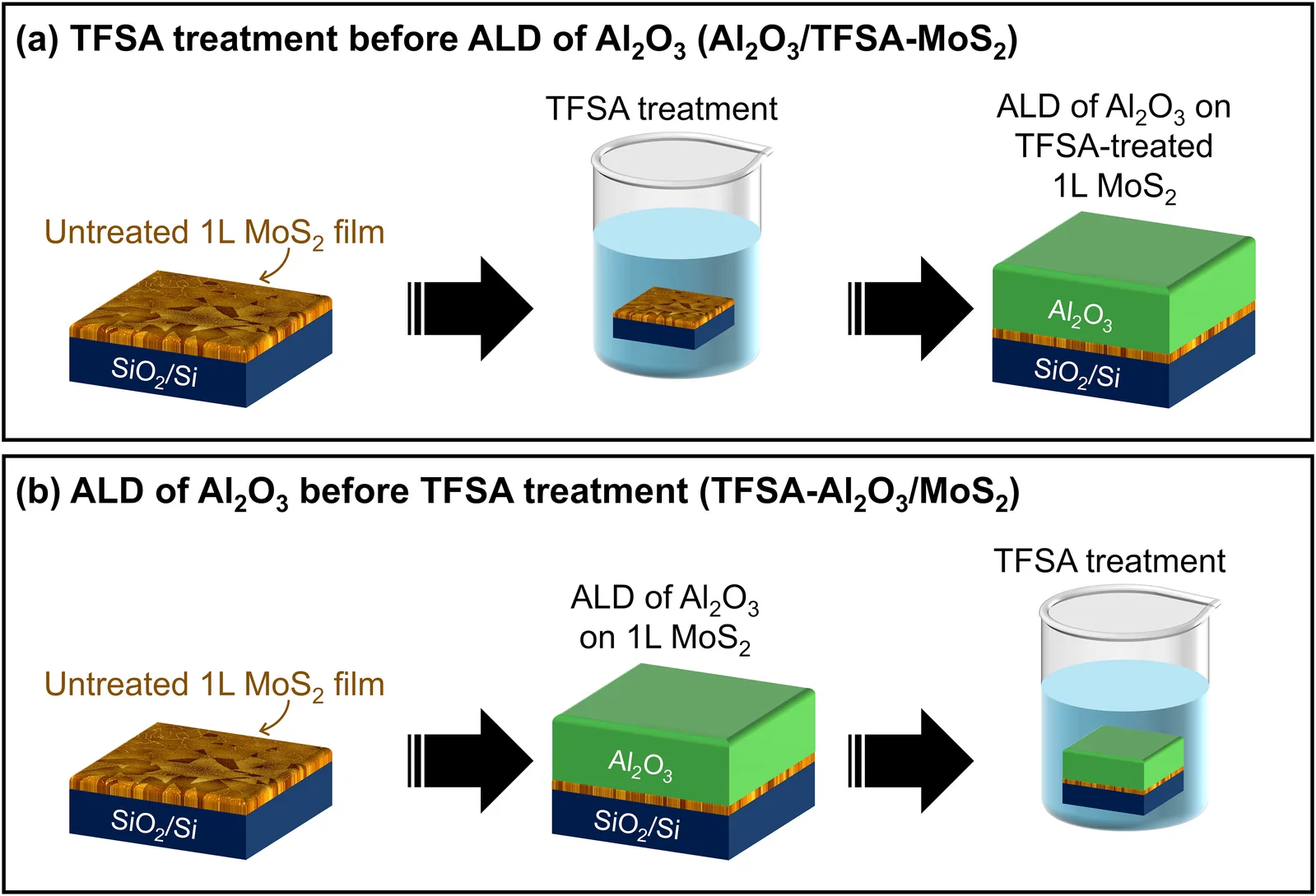
Simplified schematic illustration of the two different processing sequences investigated in this work: (a) TFSA treatment of 1L MoS_2_ prior to thermal ALD of Al_2_O_3_ and (b) thermal ALD of Al_2_O_3_ on 1L MoS_2_ followed by subsequent TFSA treatment.

In this work, we investigate how the processing order of superacid treatment and dielectric integration impacts the optical properties of 1L MoS_2_, with the two treatment routes depicted in Fig. 1. We demonstrate the instability of TFSA-enhanced 1L MoS_2_ PL emission to ALD of a dielectric overlayer. We then reverse the processing order, applying TFSA treatment to dielectric-covered 1L MoS_2_ to assess whether this encapsulation/passivation strategy can enhance the optical properties of MoS_2_. Using Al_2_O_3_ as a representative ALD-grown high-*κ* dielectric, we vary the number of ALD cycles prior to TFSA exposure to evaluate how the evolving dielectric morphology influences the overall 1L MoS_2_ PL character. We demonstrate TFSA treatment is sufficient to overcome the modified PL emission following ALD-Al_2_O_3_ growth, with substantial PL enhancement observed even at 96% dielectric coverage. This study provides the first systematic assessment of the overall impact of combined ALD-dielectric growth and superacid treatment on the optical properties of 1L MoS_2_.

## 2. Methods and materials

### Materials

A 1L MoS_2_ film (>99%) grown on a SiO_2_/Si substrate *via* atomic pressure chemical vapour deposition (APCVD) was obtained from 2D Semiconductors. The 1 × 1 cm^2^ film was received in vacuum-sealed packaging, before being cleaved into nine smaller pieces of approximately equal size, with the cleaved samples stored in a vacuum desiccator prior to treatment.

### Chemical treatment

TFSA (≥ 95.0%) and pentane (> 99%) were acquired from Sigma-Aldrich. A solution of TFSA in anhydrous pentane was prepared at a concentration of 2 mg ml^−1^. Pentane was selected as the solvent as it is known to enable substantial TFSA-induced PL enhancement in TMDCs,^27,43^ while avoiding the long-term safety concerns associated with more toxic and potentially carcinogenic alternatives, such as dichloromethane (DCM).^44,45^ TFSA was weighed out in a high-specification filtered and sealed MBRAUN UNIlab modular glovebox with gas purification system, controlled low-humidity atmosphere (< 0.1 ppm of oxygen O_2_ and water (H_2_O)), and solvent system. The TFSA–pentane solution preparation was performed in a lower specification glovebox with ambient flowing nitrogen (N_2_, relative humidity <25%). Each 1L MoS_2_ sample was immersed separately in a TFSA–pentane solution for 10 min at room temperature before being removed and allowed to dry in ambient air for 24 h prior to measurement or further processing, ensuring evaporation of the pentane solvent and effective interaction between the TFSA molecules and the MoS_2_.^25,27^

### Atomic layer deposition of Al_2_O_3_

To prevent contamination of the ALD system by superacid and solvent species, Al_2_O_3_ growth on TFSA-treated 1L MoS_2_ was performed in a separate ALD system to that used for deposition on untreated samples for subsequent TFSA processing. Thermal ALD of Al_2_O_3_ on superacid-treated 1L MoS_2_ was conducted at 150 °C in an Anric Technologies AT400 system. Trimethylaluminium (TMA) and H_2_O served as the precursor and co-reactant, respectively, with the delivery lines maintained at 100 °C. Nitrogen (N_2_) was used as both the carrier and purging gas. Table 1 summarises the reaction conditions used in this ALD process. The vacuum and thermal effects of ALD processing were also investigated using this system.

**Table 1:** Precursor and co-reactant conditions used in ALD of Al_2_O_3_ films on TFSA-treated and untreated 1L MoS_2_

Condition	TFSA/ALD	ALD/TFSA
Deposition temperature (°C)	150	200
Precursor	TMA	TMA
Number of precursor pulses in half-cycle	3	1
Precursor pulse duration (s)	0.55	0.06
Precursor purge duration (s)	13	8
Co-reactant	H_2_O	H_2_O
Number of co-reactant pulses in half-cycle	2	1
Co-reactant pulse duration (s)	0.55	0.06
Co-reactant purge duration (s)	15	8

The Al_2_O_3_ films deposited prior to TFSA treatment were grown *via* thermal ALD at 200 °C in a Veeco Fiji G2 system. Here, TMA and H_2_O were again used as the precursor and co-reactant, respectively, while argon (Ar) was employed as the carrier and purging gas. The precursor delivery lines were heated to 150 °C. The pulse and purge times associated with this Al_2_O_3_ growth process are outlined in Table 1.

### Atomic force microscopy

A Bruker Dimension Icon in the PeakForce Tapping Mode with a ScanAsystAir tip (with a nominal tip length of 115 µm, a tip radius of 2 nm and, a spring constant of 0.4 N m^−1^) was used to acquire atomic force microscopy (AFM) images of surface topographies.^46^ All AFM images comprised 256 lines per scan and were obtained at a scan rate of 0.5 Hz. Image processing and analysis was performed in the Gwyddion 2.68 software.^47^ The Fiji distribution of the ImageJ software package was used to estimate the surface coverages of the dielectric films by converting the AFM images to 8-bit grayscale and then binary images *via* the software's thresholding algorithm.^48^

### Raman and PL spectroscopy

All Raman and PL measurements were acquired at room temperature using a Renishaw inVia Reflex Raman microscope in standard confocal mode with a 50× Leica objective (numerical aperture of 0.75). A 532 nm excitation laser operated at 0.1% of maximum power (18 µW) and an 1800 lines per mm grating was used for all measurements. Single-site Raman spectra were collected as the sum of four 5 s accumulations and fitted with a two-peak Lorentzian function to capture the characteristic MoS_2_ E^1^_2g_ and A_1g_ Raman features. Corresponding single-point PL data were acquired as the sum of four accumulations of 10 s each and deconvoluted *via* multipeak Lorentzian fitting, with the exciton and trion peak energies constrained to approximate initial estimates. Raman and PL maps were collected over an area of 12 × 12 µm^2^, with a step size of 0.5 µm. A superposition of two Lorentzian peaks was used to fit the Raman spectrum at each point in the mapping data, while each mapped PL spectrum was fitted with a single Lorentzian function and the maximum absolute intensity, corresponding peak energy, and spectral linewidth extracted. Identical acquisition conditions were maintained before and after each treatment, with measurements performed at the same location on each sample. For single-spot Raman and PL measurements, the uncertainty in the location of the measurement site was typically smaller than the laser spot size (∼2 µm). The Renishaw WiRE 3.1 software package was used to collect all data and any cosmic-ray artefacts removed from the spectra where necessary.

## 3. Results and discussion

### Characterisation of untreated CVD-1L MoS_2_

**Fig. 2:**
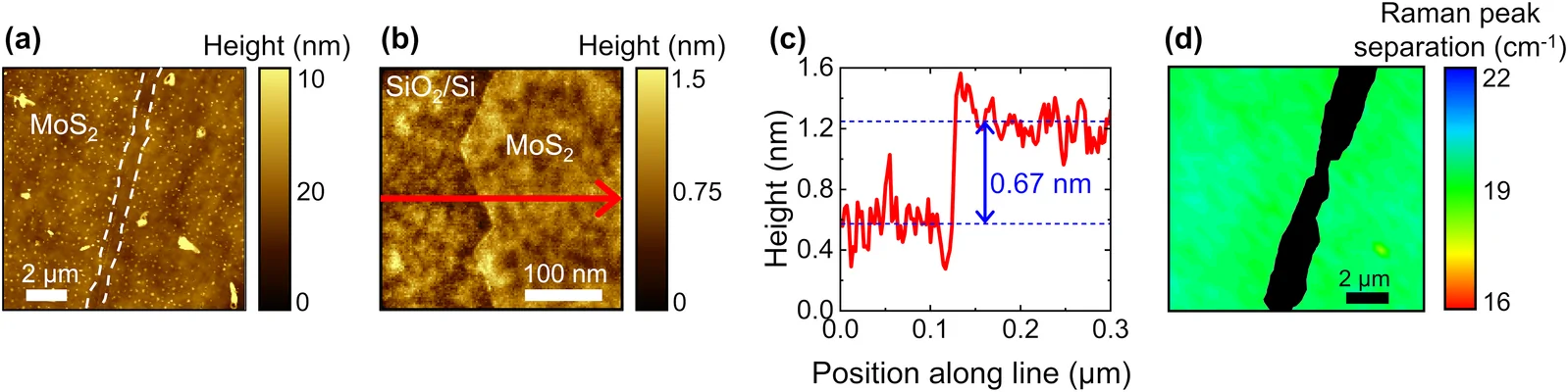
Representative characterisation of untreated CVD-1L MoS_2_. (a) Low- and (b) high-magnification topographic AFM images of a region of a CVD-1L MoS_2_ film on SiO_2_/Si. (c) Height profile measured along the red arrow in (b). (d) Map of the Raman peak separation measured from the region of untreated 1L MoS_2_ shown in (a). The black area represents the SiO_2_/Si substrate where no MoS_2_ Raman peaks are detected.

We first characterise the CVD-1L MoS_2_ film prior to any external treatment. We employ AFM imaging to verify the monolayer nature of MoS_2_. Fig. 2a presents a relatively low-magnification AFM image of a region of the MoS_2_ film containing a thin void that reveals the underlying SiO_2_/Si substrate. A higher-magnification AFM image of the boundary between the MoS_2_ and SiO_2_ regions is displayed in Fig. 2b. As shown in Fig. 2c, we estimate the step height between the MoS_2_ and adjacent SiO_2_/Si substrate to be 0.67 nm, consistent with the expected thickness of CVD-grown 1L MoS_2_.^49,50^ We utilise spatially resolved Raman spectroscopy to further confirm the presence of 1L MoS_2_. The Raman spectrum of MoS_2_ comprises two distinct, characteristic peaks: an in-plane E^1^_2g_ vibration at ∼384 cm^−1^ and out-of-plane A_1g_ feature at ∼403 cm^−1^.^51^ The separation between these two modes is related to the number of MoS_2_ layers, with a value of ∼18–21 cm^−1^ typically indicative of a single layer. Fig. 2d presents a map of the Raman peak separation obtained from the region of the MoS_2_ film shown in Fig. 2a. We find a homogenous distribution of the Raman peak separation (mean ∼19.6 ± 0.2 cm^−1^) across the surface, confirming widespread 1L MoS_2_.

### ALD-dielectric growth on TFSA-treated 1L MoS_2_

**Fig. 3:**
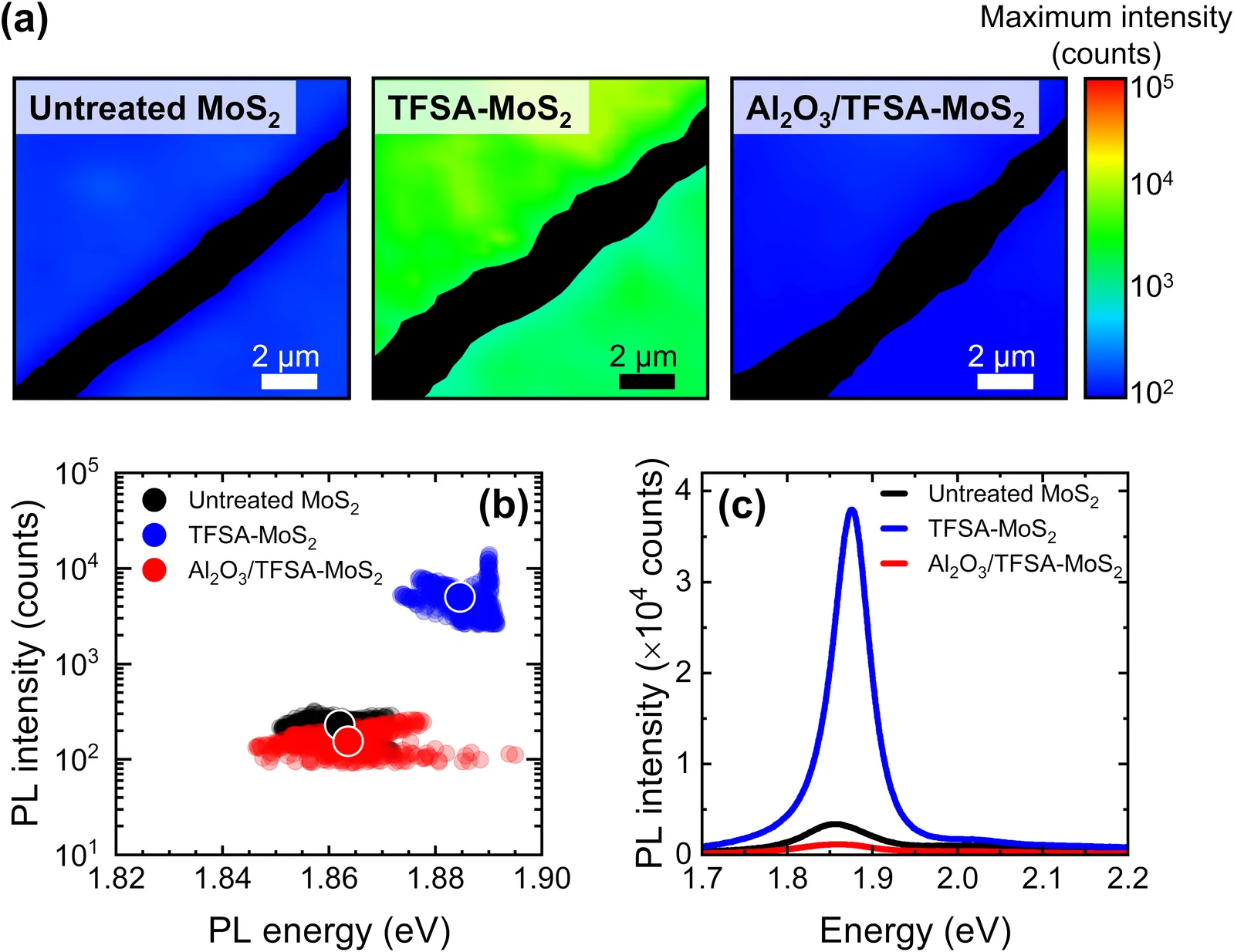
Effect of thermal ALD of Al_2_O_3_ on PL emission from TFSA-treated 1L MoS_2_. (a) Maps of maximum absolute PL intensity measured from 1L MoS_2_ before and after TFSA treatment, and after subsequent ALD of Al_2_O_3_. The black areas represent the SiO_2_/Si substrate where no MoS_2_ PL signal is detected. (b) Peak PL intensity as a function of the corresponding peak PL energy. The small translucent markers arise from every pixel extracted from each relevant PL map, with the larger solid circles indicating each calculated mean. The logarithmic scale to account for the large increase in PL intensity following TFSA treatment should be noted in (a) and (b). (c) PL spectra measured from the same single spot location on 1L MoS_2_ before and after TFSA treatment, and after subsequent ALD of Al_2_O_3_.

We next evaluate the stability of the enhanced PL character of TFSA-treated CVD-1L MoS_2_ during ALD of a dielectric. Mapping and single-site PL data measured from 1L MoS_2_ before and after TFSA treatment and following subsequent ALD of Al_2_O_3_ are presented in Fig. 3. A scatter plot of PL intensity as a function of PL energy extracted from the mapping data is displayed in Fig. 3b (full spatial maps of the PL energy and full width at half maximum (FWHM) are provided in Fig. S1 in the SI, with a magnified view of the low-intensity region of Fig. 3b displayed in Fig. S2).

Initial TFSA processing yields the expected increased, blueshifted, and sharpened PL emission, with an observed enhancement of one order of magnitude consistent with our recent report of TFSA–pentane treatment.^27^ The spectral sharpening indicates increased exciton lifetime and reduced non-radiative recombination.^52,53^ The modified PL character is associated with the synergistic combination of S vacancy passivation (predominantly by dissociated SO_2_-based species) and p-type doping by electronegative fluorine (F) atoms in TFSA.^17,23,24,54,55^ The TFSA-MoS_2_ interaction proceeds *via* the spontaneous formation of a thin superacid “nanolayer” on the MoS_2_ surface as the solvent evaporates during the drying process.^25,27,56^ ALD of Al_2_O_3_, however, removes the elevated PL emission, with the 1L MoS_2_ PL intensity reduced by ∼33% relative to that emitted prior to any treatment. The PL peak energy is also redshifted back to ∼1.86 eV, while the spectral linewidth is broadened to ∼112 meV, comparable to that of untreated MoS_2_. An AFM image of the Al_2_O_3_ film deposited on TFSA-treated 1L MoS_2_ after 200 ALD cycles is provided in Fig. S3 in the SI, showing the presence of dielectric islands in an incompletely coalesced layer. It is clear that thermal ALD growth of this Al_2_O_3_ layer negates the optical improvement in 1L MoS_2_ induced by TFSA treatment.

To probe changes to the excitonic contributions, we performed multipeak Lorentzian fitting of the single-site PL spectra to separate the constituent spectral features, with full deconvolutions provided in Fig. S4 in the SI. The PL signal from untreated 1L MoS_2_ comprised a dominant negatively charged trion, A^−^, feature at ∼1.85 eV, and A and B excitonic transitions at ∼1.87 eV and ∼2.0 eV, respectively. The ratio between the absolute intensities of the A^−^ and A peaks, A^−^/A, can be related to the electron density in 1L MoS_2_, with a larger excess of electrons yielding a higher A^−^/A ratio.^57^ As expected, the initial TFSA treatment suppressed the trion contribution, significantly reducing the A^−^/A ratio from ∼1.9 to ∼0.1, evidence of a p-type doping effect.^23,24,27^ Subsequent ALD growth of Al_2_O_3_ then increased the A^−^/A ratio to ∼1.6, indicating increased trion formation and near-complete reversal of the p-type doping effects of TFSA treatment. The PL spectrum can also reveal information on the abundance of defects, with the ratio of the absolute intensity of the B exciton peak to that of the A exciton known to be a reliable indicator of defect state density.^58^ We employed a three-peak deconvolution of the PL spectra, and so defined the excitonic intensity ratio as B/(A + A^−^). In agreement with previous reports,^23,27^ TFSA treatment induces a significant reduction in the relative contribution of the B exciton, decreasing the B/(A + A^−^) ratio from ∼0.2 to near zero, consistent with effective defect passivation. After ALD of Al_2_O_3_ on TFSA-treated 1L MoS_2_, the B/(A + A^−^) ratio increased back to ∼0.2. We therefore suggest that ALD growth of Al_2_O_3_ increases the defect density in superacid-treated MoS_2_, arising either from the reintroduction of previously passivated vacancies or *via* the formation of new defective sites, possibly at the MoS_2_/dielectric interface. However, since the FWHM and B/(A + A^−^) ratio both returned to values comparable to those typical of untreated MoS_2_, we speculate that reactivation of intrinsic S vacancies is the primary defect creation process that occurs during ALD of Al_2_O_3_ on TFSA-treated 1L MoS_2_. We suggest that Al_2_O_3_ growth does not primarily generate new defect types but instead disrupts or removes the adsorbed superacid “nanolayer” that facilitates passivation and charge transfer. Hence, the TFSA-induced improvement in PL behaviour is not stable under ALD processing. The dielectric growth reverses both the defect suppression and p-type doping effects induced by interaction with the superacidic TFSA.

### Isolation of ALD processing effects on TFSA-treated 1L MoS_2_

**Fig. 4:**
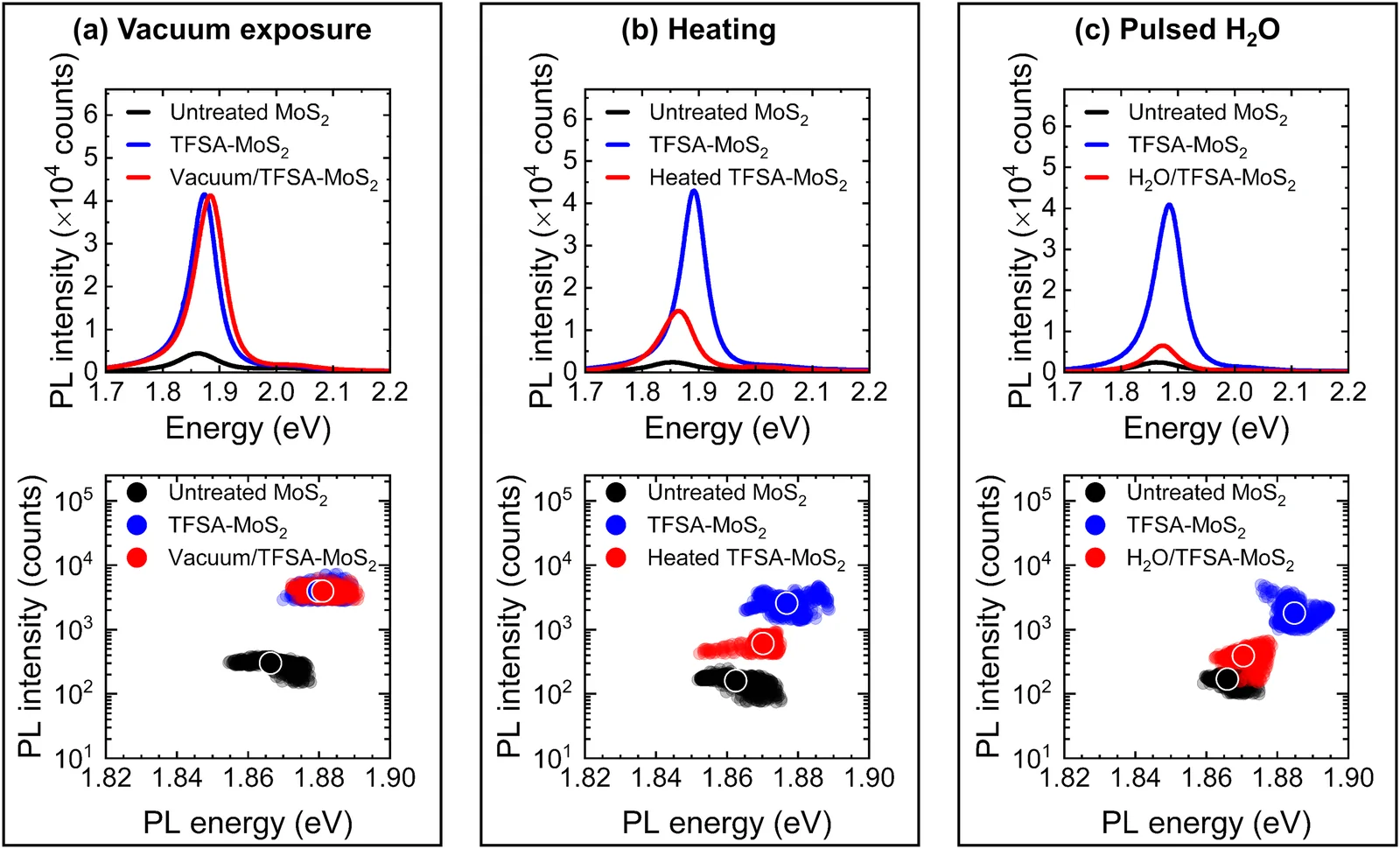
Single-site PL spectra (top) and scatter plots of peak PL intensity as a function of the corresponding peak PL energy extracted from PL mapping data (bottom) measured from 1L MoS_2_ before and after TFSA treatment, and after subsequent (a) ALD-vacuum exposure, (b) heating at 150 °C in the ALD chamber, and (c) ALD-H_2_O treatment. No TMA precursor was pulsed during any of these three processes. In the scatter plots, the small translucent markers arise from every pixel extracted from each relevant PL map, with the larger solid circles indicating each calculated mean average. The logarithmic scale to account for the large increase in PL intensity following TFSA treatment should be noted.

To ascertain the origin of the reduced PL emission observed following dielectric growth, we subjected separate TFSA-treated 1L MoS_2_ samples to several modified ALD processes designed to isolate the effects of vacuum exposure, thermal heating, and H_2_O co-reactant pulsing in the absence of precursor exposure. We have previously reported that the vacuum environment, thermal processing, and pulsed exposure to H_2_O during ALD-dielectric growth have negligible impacts on the 1L MoS_2_ optical properties.^23^ However, the sensitivity of superacid-treated MoS_2_ to these individual processing steps has not been previously established. Here, we treated a cleaved 1L MoS_2_ sample with TFSA before placing it in the ALD chamber under vacuum (chamber pressure <15 mTorr) for ∼2 h, a period comparable to the duration of a 200-cycle deposition. A separate TFSA-treated 1L MoS_2_ sample was placed in the ALD chamber for ∼2 h, with the chamber pumped to vacuum, and all heaters set to the temperatures typical of a 150 °C deposition, but without the introduction of any precursor gases. A third TFSA-enhanced 1L MoS_2_ sample was subjected to a modified ALD-Al_2_O_3_ recipe that cycled only H_2_O pulses, omitting the TMA precursor injection steps but retaining the vacuum and thermal conditions characteristic of a standard growth process. Single-spot PL spectra and scatter plots associated with each of these processing routes are shown in Fig. 4, with the associated PL mapping data and selected magnified views of the scatter plots provided in Fig. S5–S8 of the SI.

In each case, the initial TFSA treatment yielded the expected enhanced MoS_2_ PL character, and this elevated PL emission survived exposure to vacuum in the ALD chamber for ∼2 h (Fig. 4a), with negligible changes to the PL intensity, energy, and spectral linewidth. However, heating to 150 °C under vacuum for ∼2 h (Fig. 4b) reduced the PL intensity from TFSA-treated 1L MoS_2_ by ∼4 ×. This heating under vacuum also induced an appreciable redshift of ∼7 meV in the PL peak energy relative to the blueshifted position observed after TFSA treatment, accompanied by noticeable spectral broadening to a FWHM intermediate between that of untreated and TFSA-treated 1L MoS_2_. Pulsed exposure to H_2_O (Fig. 4c) produced a more pronounced modification of the TFSA-enhanced MoS_2_ PL signal, resulting in a five-fold attenuation of the intensity, with a redshift of ∼14 meV and a ∼1.3× increase in the FWHM also observed. Such changes to the PL signal indicate partial reversal of the TFSA-induced p-type doping and defect passivation, suggesting that the thermal effects and exposure to H_2_O during ALD processing can destabilise or weaken the interaction between TFSA-derived species and the 1L MoS_2_ surface. Compared to the complete removal of the TFSA-induced PL enhancement observed following ALD of Al_2_O_3_, the PL intensity remains partially elevated after either heating under vacuum or H_2_O pulsing. We therefore conclude that the overall impact of Al_2_O_3_ deposition on TFSA-treated 1L MoS_2_ arises from the combined effects of thermal processing and interaction with reactive precursor and co-reactant species.

### TFSA treatment of dielectric-encapsulated 1L MoS_2_

**Fig. 5:**
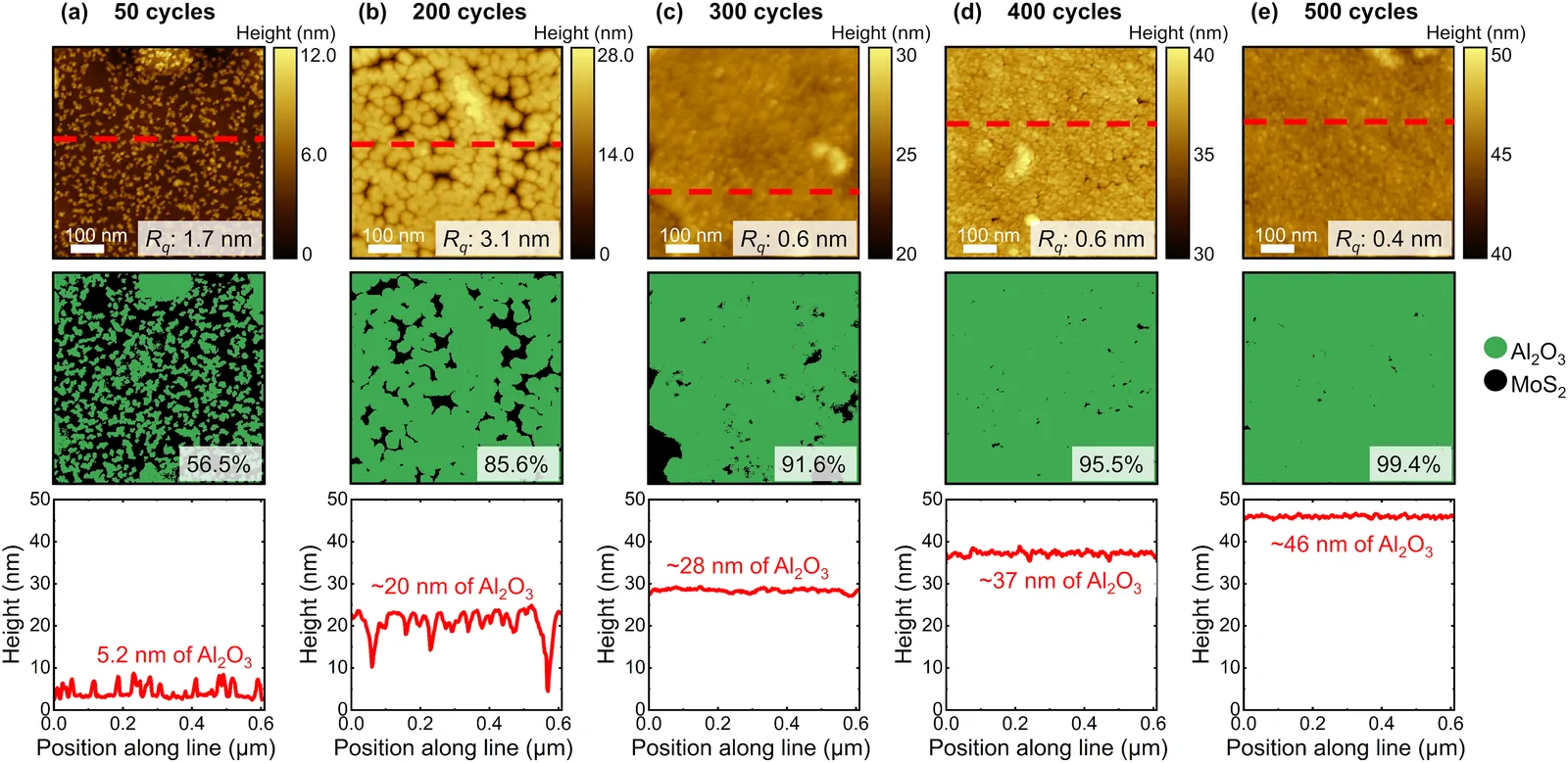
High-magnification AFM images (top), maps of estimated surface coverage extracted *via* ImageJ^48^ (middle), and representative line profiles (bottom) of the ALD-Al_2_O_3_ films grown on 1L MoS_2_ at cycle numbers (a) 50, (b) 200, (c) 300, (d) 400, and (e) 500. The root mean square (RMS) surface roughness, *R*_*q*_, is indicated in each image. The line profiles are traced along the red dashed lines in each AFM image and the average island height or estimated dielectric thickness is indicated on the bottom row. The height trace of each AFM image is offset such that the mean value of each line profile is centred on the expected thickness of Al_2_O_3_ at each cycle number, using the growth characteristics on CVD-1L MoS_2_ evaluated in our previous study.^32^

Having demonstrated superacid treatment of 1L MoS_2_ prior to ALD of Al_2_O_3_ does not yield overall PL enhancement, we now explore exposing Al_2_O_3_-encapsulated MoS_2_ to TFSA in an encapsulation/passivation approach. We grew several Al_2_O_3_ films on separate 1L MoS_2_ samples, varying the number of ALD cycles, from 50 cycles to 500 cycles. Fig. 5 displays selected high-magnification AFM images of the Al_2_O_3_ film grown at each number of cycles, with approximated maps of the dielectric surface coverage and representative line profiles also displayed for each image. At least five different regions of each sample were probed, with representative images presented here. While AFM obtains the relative height variation, we offset the height trace for each sample such that the mean value of the line profile is centred on the Al_2_O_3_ thickness estimated *via* the growth characteristics determined in our previous study.^32^

Dielectric surface coverage increased systematically with ALD cycle number, from only ∼57% at 50 cycles to ∼96% at 400 cycles. Near-complete surface coverage (>99%), with minimal density of significant voids or pinholes observed in the deposited Al_2_O_3_ film, was achieved only after 500 cycles. Each Al_2_O_3_-encapsulated 1L MoS_2_ sample was then submerged separately in a TFSA-pentane solution, before being removed and dried in air. To verify the ALD-grown Al_2_O_3_ films survived exposure to TFSA, high-magnification AFM images were acquired from each Al_2_O_3_-covered 1L MoS_2_ sample following superacid treatment, as displayed in Fig. S9 in the SI. No significant changes to the dielectric film morphologies were observed after TFSA processing, demonstrating the robustness of each Al_2_O_3_ film to the chemical treatment.

**Fig. 6:**
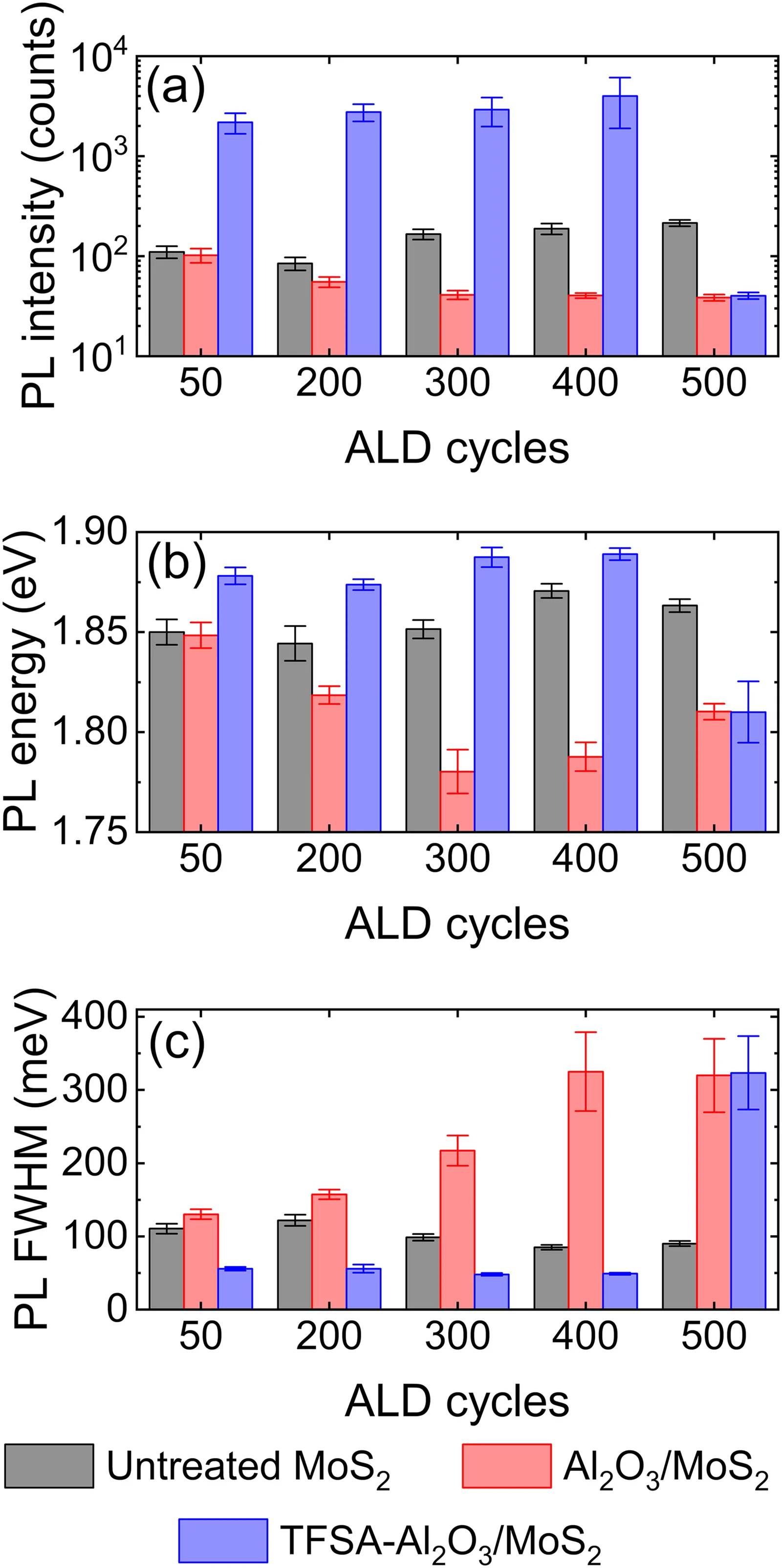
Mean (a) PL intensity, (b) PL energy, and (c) PL FWHM as averaged from mapping data measured from 1L MoS_2_ before (black) and after (red) ALD of Al_2_O_3_, and after subsequent TFSA treatment (blue) for each number of ALD cycles. Full PL mapping data can be found in Section S8 of the SI. The error bars correspond to one standard deviation in each calculated mean value. The logarithmic scale to account for the large increase in PL intensity following TFSA treatment should be noted in (a).

**Fig. 7:**
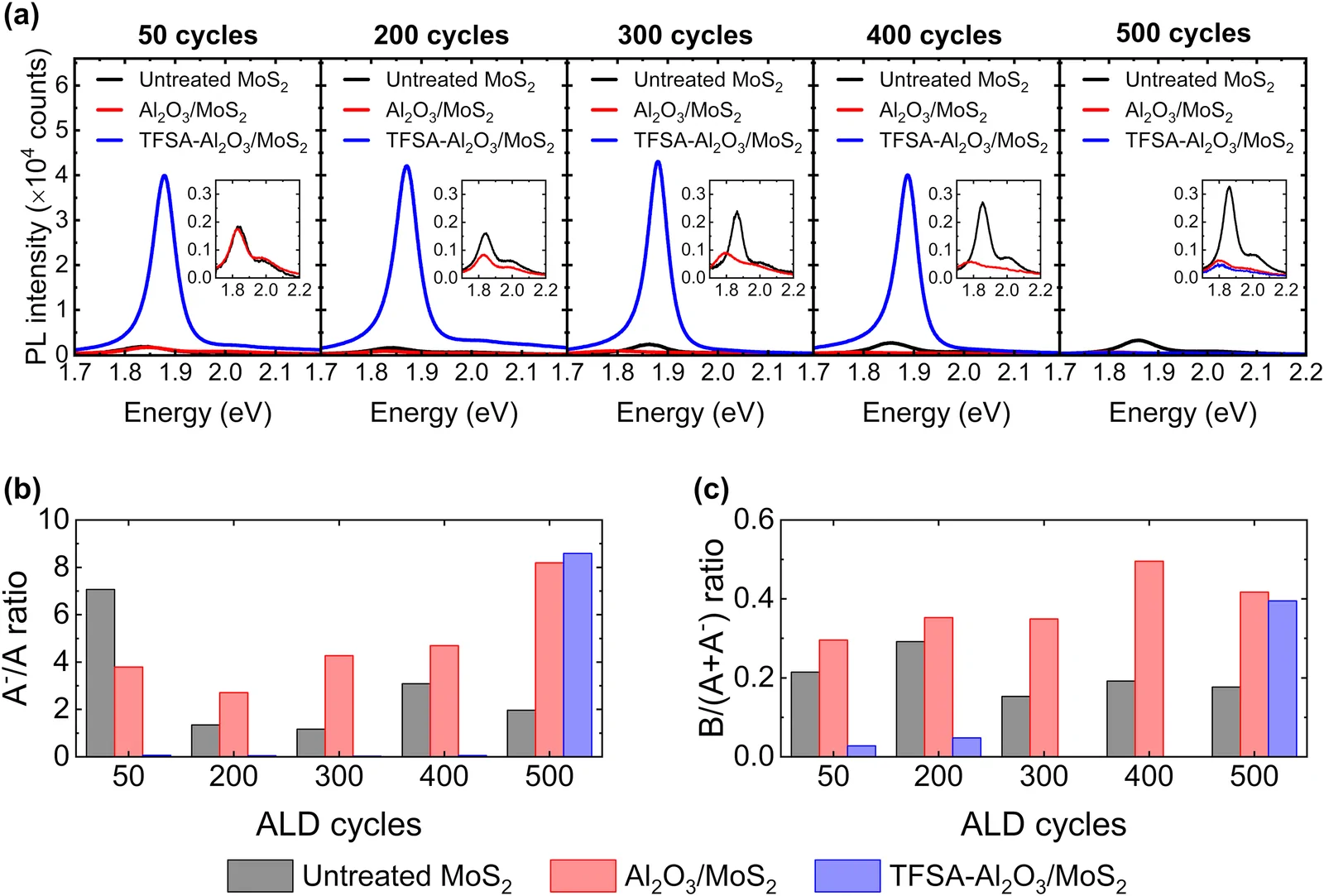
(a) Single-site PL spectra measured from each 1L MoS_2_ sample before (black) and after (red) ALD of Al_2_O_3_, and after subsequent TFSA treatment (blue) for each number of ALD cycles. A magnified view of the low intensity spectra is displayed in each inset. (b) A^−^/A and (c) B/(A + A^−^) intensity ratios extracted from each of the spectra in (a). Full deconvoluted single-site PL spectra are provided in Fig. S15 in the SI.

The PL emission from each Al_2_O_3_-encapsulated sample was measured before and after ALD of Al_2_O_3_, and following TFSA treatment. Fig. 6 presents a comparison of the intensity, energy, and FWHM averaged from the PL mapping data acquired after each processing step, with representative single-site PL spectra displayed in Fig. 7. Full PL maps and deconvoluted single-site spectra are provided in Section S8 (Fig. S10–S14) and Fig. S15 of the SI, respectively. As expected, and as previously reported,^23,32^ ALD growth of Al_2_O_3_ on 1L MoS_2_ attenuates, redshifts, and broadens the PL signal. The extent of these modifications progresses with ALD cycle number, with accompanying increases in the A^−^/A (Fig. 7b and B)/(A + A^−^) (Fig. 7c) ratios consistent with increased interfacial n-type doping and heightened defect-state densities as Al_2_O_3_ approaches complete coverage.

Following TFSA treatment of the Al_2_O_3_-encapsulated samples, a strong recovery and further substantial enhancement of the PL emission was observed for all ALD cycle numbers up to 400 cycles (Fig. 6a). Each increased PL intensity was accompanied by a blueshift in the PL energy (Fig. 6b) and a narrowing of the PL FWHM (Fig. 6c), consistent with defect passivation and p-type doping.^23,27^ We define a PL enhancement factor as the ratio of the maximum absolute PL intensity averaged from each TFSA-treated Al_2_O_3_/1L MoS_2_ sample to that obtained from the corresponding encapsulated sample before superacid exposure. An analogous sharpening factor is also introduced to evaluate changes to the PL spectral width. At 50 ALD cycles, TFSA treatment induced a ∼20× PL enhancement, a ∼30 meV blueshift, and a ∼2× narrowing of the PL linewidth relative to the Al_2_O_3_-covered state. The degree of PL enhancement increased progressively with ALD cycle number up to ∼100× at 400 cycles. The magnitudes of blueshift and FWHM sharpening also increased systematically with cycle number; at 400 ALD cycles, the relative blueshift in PL energy was ∼100 meV and the FWHM was reduced significantly (by ∼7 ×). The increasing PL modification with cycle number is consistent with the introduction of additional ALD-induced defects that may subsequently be passivated by TFSA. Dielectric deposition can create a variety of defect states in 1L MoS_2_,^32^ including S vacancies.^59^ TFSA is known to repair intrinsic S vacancies effectively and may similarly passivate such process-induced defects. As a result, non-radiative recombination pathways are suppressed and the PL emission becomes exciton-dominated, reversing the ALD-induced n-type doping, as evidenced by the near-zero A^−^/A and B/(A + A^−^) intensity ratios observed after TFSA treatment (Fig. 6b and c). At 500 ALD cycles, however, the PL intensity was not enhanced by the TFSA treatment, remaining at a magnitude comparable to that emitted after ALD of Al_2_O_3_, with the PL energy and FWHM also unchanged by superacid exposure. In addition, the A^−^/A and B/(A + A^−^) ratios were also unaffected, demonstrating the persistence of strong n-type doping and a high defect density.

**Fig. 8:**
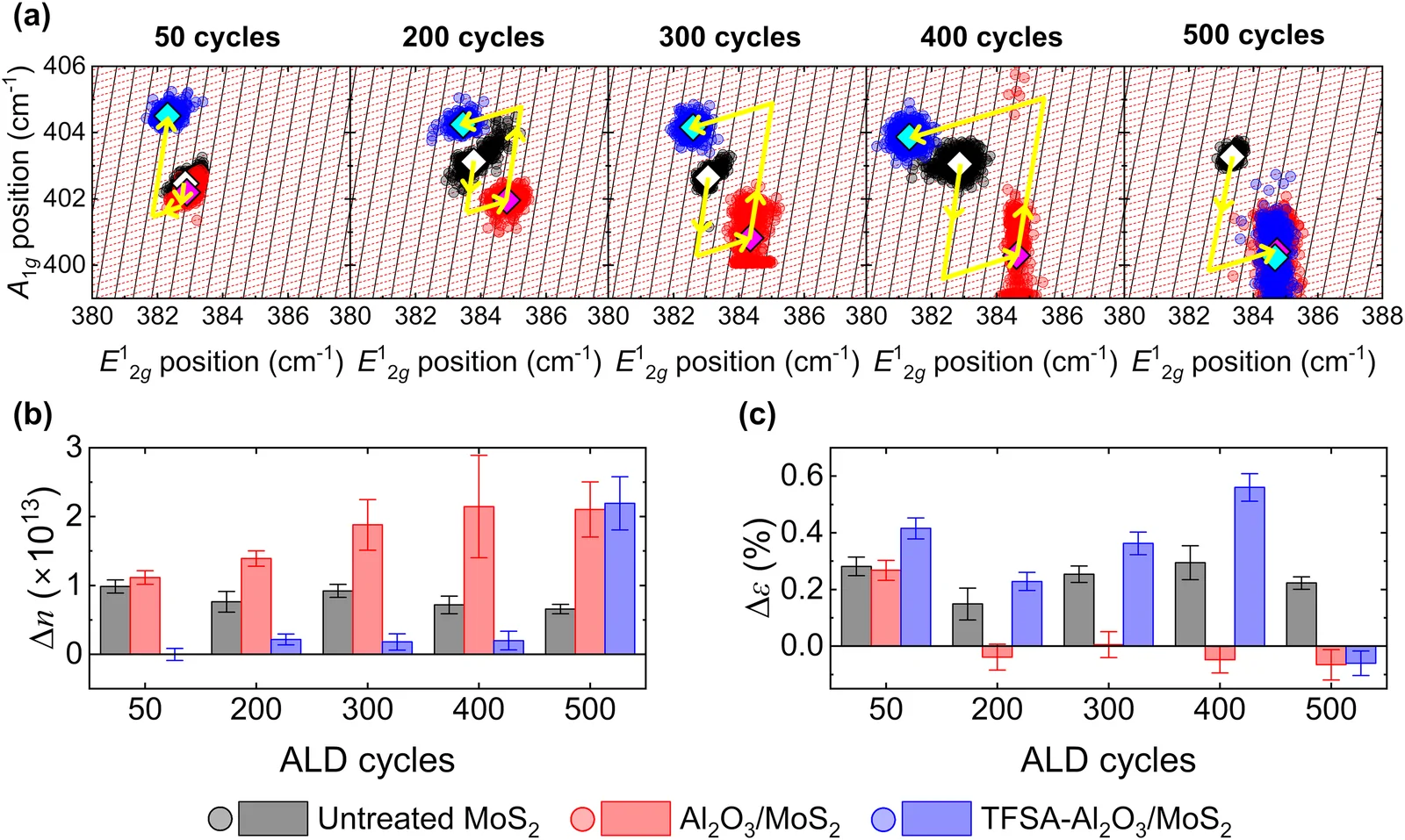
(a) Correlative plots of A_1g_ and E^1^_2g_ peak positions extracted from Raman mapping data acquired from each 1L MoS_2_ sample before (black) and after (red) ALD of Al_2_O_3_, and after subsequent TFSA treatment (blue) for each number of ALD cycles. The small circular translucent markers arise from every pixel extracted from the Raman mapping data, and the large diamond points indicate the corresponding average values. Strain isolines (black solid lines) indicate Δ*ε* = ±0.1% variations in the strain and doping isolines (red dashed lines) correspond to relative changes in the electron density of Δ*n* = ± 0.1 × 10^13^ cm^−2^. Full Raman mapping data can be found in Section S10 of the SI. Mean relative (b) electron density (Δ*n*) and (c) mechanical biaxial strain (Δ*ε*) extracted from (a). The error bars correspond to one standard deviation in each calculated mean value.

Using spatially resolved Raman spectroscopy, we further elucidate the underlying mechanisms behind modification of the optical properties of 1L MoS_2_ following ALD of Al_2_O_3_ and subsequent TFSA treatment. Spatial maps of the Raman peak positions are displayed in Section S10 (Fig. S16–S20) in the SI. We performed a correlative analysis of Raman mapping data to separate changes to the mechanical strain, *ε*, and electron density, *n*, in 1L MoS_2_. Such quantitative evaluation of strain and doping is commonly applied to a range of 2D materials,^23,32,36,60–67^ and full details of this process are provided in Section S11 of the SI. Resulting plots of A_1g_ peak position as a function of E^1^_2g_ wavenumber for each number of ALD cycles are shown in Fig. 8, each overlain with a grid of strain (black) and doping (red dashed) isolines.

The extracted variations in electron density (Fig. 8b) and strain (Fig. 8c) are in excellent agreement with the PL analysis discussed above. At all cycle numbers, ALD of Al_2_O_3_ increased the electron density, consistent with the trend in the A^−^/A intensity ratio and indicating progressive n-type doping. Subsequent TFSA treatment substantially reduced the electron density in Al_2_O_3_-encapsulated 1L MoS_2_ samples for all ALD cycle numbers up to 400 cycles. This confirms the effective reversal of ALD-induced doping *via* charge transfer to TFSA species and suppression of electron-rich defective sites.^68^ In contrast, TFSA treatment of the 1L MoS_2_ sample coated with 500 ALD cycles of Al_2_O_3_ had little effect on the electron density, which remained relatively high at ∼2 × 10^13^ cm^−2^.

Mechanical strain in 1L MoS_2_ is known to be sensitive to the presence of S vacancies, with heightened biaxial tensile strain associated with extrinsic occupation of such vacancy sites, corresponding to an increase in the lattice parameters.^17,24^ Following 50 cycles of Al_2_O_3_, we observed no appreciable change in the strain, consistent with low dielectric coverage. At higher cycle numbers, deposition of Al_2_O_3_ led to an increase in compressive strain in 1L MoS_2_, which we attribute to dielectric-induced mechanical interactions and interfacial bonding. Subsequent TFSA treatment of Al_2_O_3_-encapsulated 1L MoS_2_ yielded a progressive increase in mechanical tension in all samples up to 400 cycles, reaching ∼0.6% relative tensile strain at 400 cycles. This increased tensile strain is consistent with the passivation of S vacancies indicated by the changes in the PL emission, namely the narrowed linewidth and reduced B/(A + A^−^) ratio. However, it is important to note that additional factors, such as changes in the dielectric-induced interfacial strain, may also influence the overall strain in the underlying 1L MoS_2_ layer. At 500 cycles of Al_2_O_3_, however, TFSA treatment gave no increase in mechanical tension, with the small compressive strain (< 0.1%) induced by the dielectric growth maintained after superacid exposure. This absence of strain modification further supports the conclusion that TFSA is unable to influence 1L MoS_2_ through the Al_2_O_3_ layer formed after 500 ALD cycles.

We rationalise the cycle-dependent evolution of the Raman and PL responses in terms of the accessibility of the MoS_2_ surface to TFSA-derived species. TFSA-induced enhancement of uncovered 1L MoS_2_ proceeds *via* the spontaneous formation of a nanoscale superacid layer,^25,27,56^ enabling adsorption of dissociated TFSA fragments. The elevated PL intensity from TFSA-treated 1L MoS_2_ is widely attributed to the combined effects of S vacancy passivation and p-type charge transfer.^17,23,24,54,55^ In this mechanism, dissociated SO_2_-based species are thought to directly repair vacancy sites,^17^ with protonic species also able to localise at or close to such defects.^69^ Electronegative F-containing TFSA species can withdraw electron density from the MoS_2_ lattice.^23,24^ Therefore, for TFSA to enhance the PL character of Al_2_O_3_-encapsulated 1L MoS_2_, protonic, S-, and F-containing fragments must be able to reach the basal plane. At low ALD cycle numbers, the Al_2_O_3_ film is incompletely coalesced and comprises distinct islands (Fig. 5), leaving the underlying 1L MoS_2_ partially exposed through pinholes and voids. Here, the dissociated TFSA-derived species can directly access the MoS_2_ surface, enabling defect passivation and charge transfer to enhance the PL. As the number of ALD cycles increases, the Al_2_O_3_ film becomes thicker and more continuous, reducing direct access to the underlying 1L MoS_2_. TFSA species may instead reach the MoS_2_ basal plane *via* defects such as grain boundaries,^70,71^ or diffusion through the Al_2_O_3_ layer. TFSA molecules have been shown to diffuse effectively through fluoropolymeric layers to interact with underlying MoS_2_.^41^ While transport through polymers is relatively fast, the diffusion rates of species dissociated from TFSA are expected to be much slower in a higher-density amorphous oxide environment,^72^ such as that of ALD-grown Al_2_O_3_ films.^73,74^ Previous studies have shown that TFSA-derived species can be detected within oxide layers following superacid treatment,^22^ while sterically smaller dissociated species, such as protons, have been reported to migrate through ALD-grown dielectric layers.^75,76^ Since we observe the persistence of TFSA-induced enhancement at high dielectric coverages up to ∼96%, diffusion through the Al_2_O_3_ layer cannot be excluded. As the Al_2_O_3_ film becomes thicker and approaches complete coalescence, transport pathways for TFSA-derived species to the underlying 1L MoS_2_ are expected to become increasingly restricted, suppressing the efficacy of TFSA treatment. Once the Al_2_O_3_ film is sufficiently thick and near-continuous, no TFSA-induced enhancement of the underlying MoS_2_ is observed.

## 4. Conclusions

We have examined the combination of two processing routes that are critical for realisation of 1L MoS_2_-based optoelectronic devices: superacid treatment and ALD of a high-*κ* dielectric material. We have established the instability of TFSA-enhanced 1L MoS_2_ PL during subsequent ALD-dielectric growth, demonstrating that this degradation arises from thermal exposure and interaction with reactive precursor species during the deposition process. We then investigated the reverse processing sequence, exploring the superacid treatment of Al_2_O_3_-coated 1L MoS_2_ as an encapsulation/passivation approach. We revealed that, when performed after dielectric deposition, TFSA treatment can recover and significantly enhance the optical properties of dielectric-encapsulated 1L MoS_2_, even at high dielectric coverages. By systematically varying the number of ALD cycles in the growth of Al_2_O_3_, we have shown that the effectiveness of post-encapsulation superacid treatment is governed by the ability of TFSA-derived species to access the MoS_2_ surface. Strong PL enhancement, accompanied by suppressed defect signatures and reversal of n-type doping, is observed for Al_2_O_3_ films of up to ∼37 nm thickness and ∼96% surface coverage. At greater thickness and dielectric coverage, however, the Al_2_O_3_ layer is sufficiently thick and completely coalesced to eliminate both diffusion and pinhole-mediated access, preventing TFSA-induced enhancement of 1L MoS_2_. This study provides important insights for the integration of chemical passivation strategies in practical device architectures. The extension of this approach to other high-*κ* dielectrics, including HfO_2_, ZnO, and ZrO_2_, whose growth characteristics and interfacial properties on CVD-1L MoS_2_ are known to differ from those of Al_2_O_3_, represents an important direction for future work.

## Author contributions

This study was conceptualised by B. F. M. H. Experimental work was primarily conducted by B. F. M. H., with support from K. J. H. and S. L. P. Data analysis was largely performed by B. F. M. H., with discussions and contributions from S. L. P., N. E. G., and J. D. M. The manuscript was written by B. F. M. H., with input and editing from S. L. P., K. J. H., N. E. G., and J.D.M.

## Conflicts of interest

There are no conflicts to declare.

## Supplementary Material

TC-014-D6TC02199A-s001

## Data Availability

Data underpinning figures in this paper can be freely downloaded from https://doi.org/10.82444/warw.33952129. Requests for additional data should be made directly to the corresponding author. Supplementary information (SI): additional PL mapping data, AFM imaging, and single-site PL deconvolution for ALD of Al_2_O_3_ on TFSA-treated 1L MoS_2_; PL mapping data for ALD control processes on TFSA-treated 1L MoS_2_; AFM images, PL mapping data, single-site PL deconvolution, and Raman mapping data for TFSA treatment of Al_2_O_3_-encapsulated 1L MoS_2_; and correlative analysis of strain and doping in 1L MoS_2_. See DOI: https://doi.org/10.1039/d6tc02199a.
