# Supplementary material for: Superacid treatment of dielectric-encapsulated monolayer molybdenum disulfide for enhanced photoluminescence
Source: J Mater Chem C Mater. 2026 Sep 25;14(41):19992–20003. doi: 10.1039/d6tc02199a (PMC13613486; doi:10.1039/d6tc02199a)
Supplement: Supplementary file 1 [file TC-014-D6TC02199A-s001.pdf]

Supplementary Information

Superacid treatment of dielectric-encapsulated monolayer molybdenum disulfide for enhanced photoluminescence

Brendan F. M. Healy[1,*], *Kelsey J. Hall*[1], *Sophie L. Pain*[2], *Nicholas E. Grant*[1], *and John D. Murphy*[2]

[1]School of Engineering, University of Warwick, Coventry, CV4 7AL, UK

[2]School of Engineering, University of Birmingham, Edgbaston, Birmingham, B15 2TT, UK

***Corresponding Author**

Brendan F. M. Healy – Email: brendan.f.m.healy@warwick.ac.uk

Contents

S1. Additional PL mapping data for ALD-Al_2O_3 on TFSA-treated 1L MoS_2 2

S2. Magnified PL scatter plot for ALD-Al_2O_3 on TFSA-treated 1L MoS_2 2

S3. AFM image of ALD-Al_2O_3 on TFSA-treated 1L MoS_2 3

S4. Deconvolution of single-spot PL spectra for ALD-Al_2O_3 on TFSA-treated 1L MoS_2 ... 3

S5. PL mapping data for ALD control processes on TFSA-treated 1L MoS_2 4

S6. Magnified PL scatter plots for ALD control processes on TFSA-treated 1L MoS_2 7

S7. AFM images of TFSA-treated Al_2O_3/1L MoS_2 7

S8. PL mapping data for TFSA treatment of Al_2O_3-encapsulated 1L MoS_2 8

S9. Deconvolution of single-spot PL spectra for TFSA treated Al_2O_3/1L MoS_2 13

S10. Raman mapping data for TFSA treatment of Al_2O_3-encapsulated 1L MoS_2 14

S11. Correlative analysis of strain and doping in 1L MoS_2 17

References 19

S1. Additional PL mapping data for ALD-Al_2O_3 on TFSA-treated 1L MoS_2

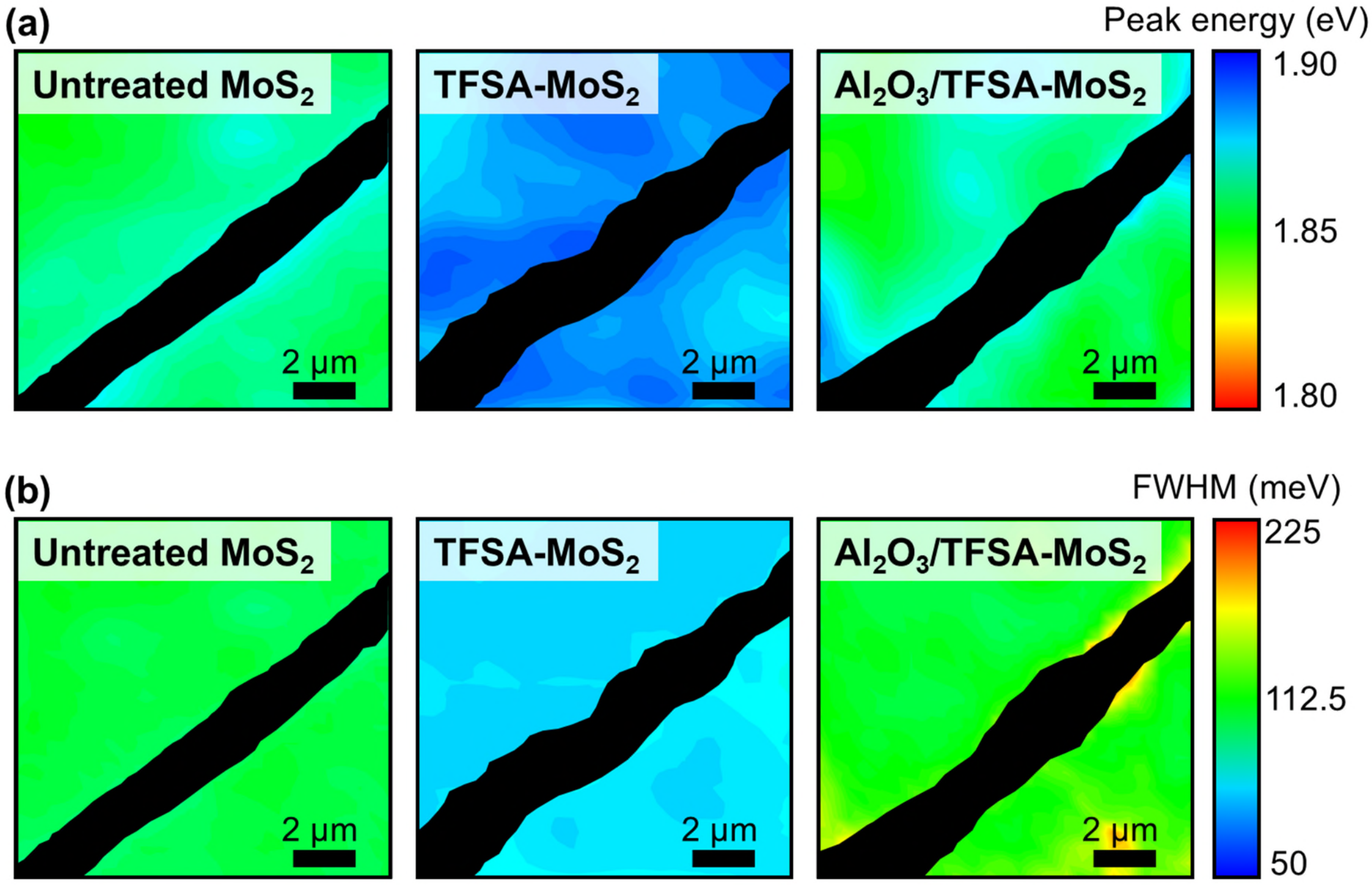

Figure S1: Maps of (a) peak PL energy and (b) PL full width at half maximum (FWHM) measured from 1L MoS_2 before and after TFSA treatment, and after subsequent thermal ALD of Al_2O_3. The black areas represent the SiO_2/Si substrate where no MoS_2 PL signal is detected.

S2. Magnified PL scatter plot for ALD-Al_2O_3 on TFSA-treated 1L MoS_2

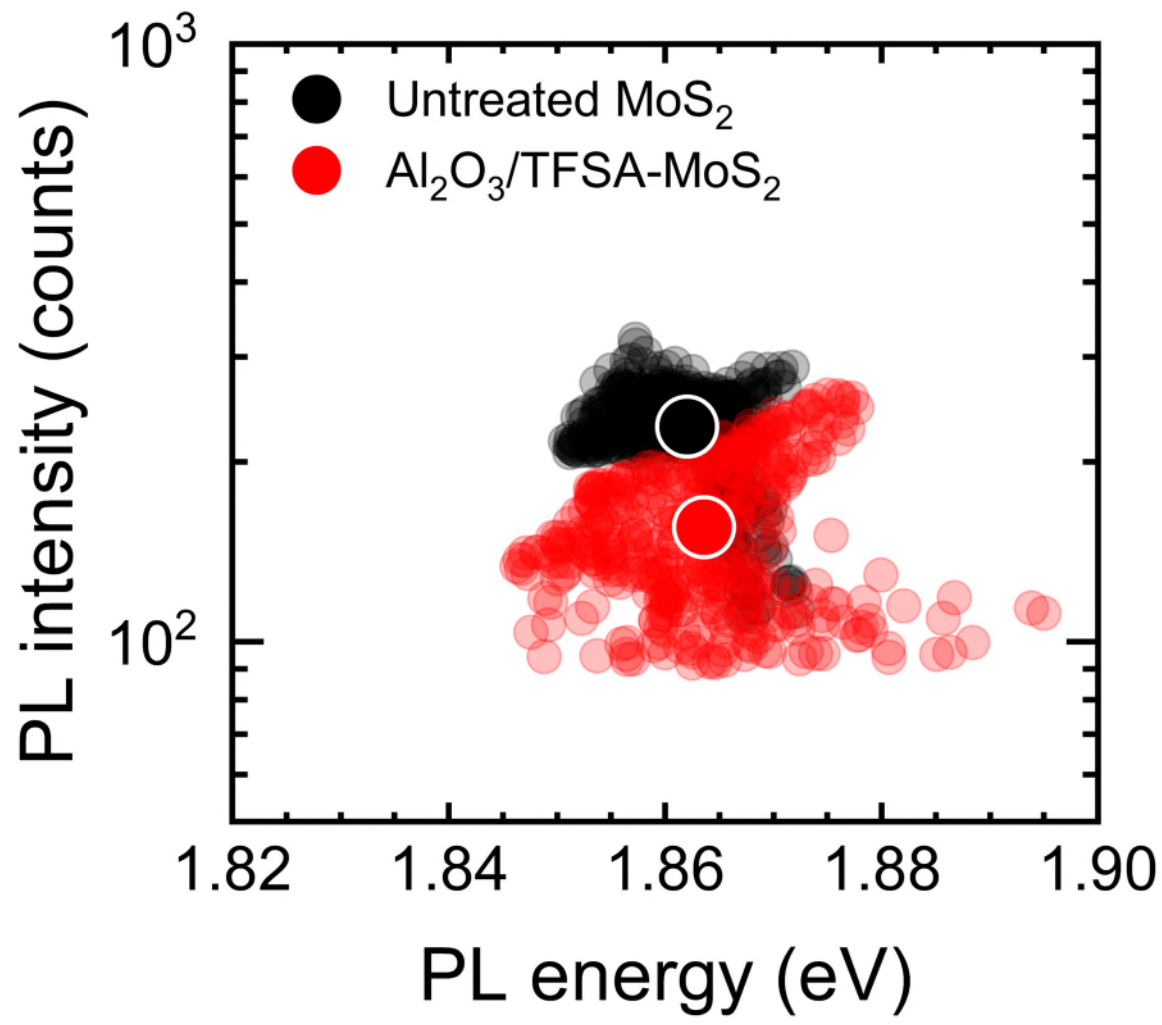

Figure S2: Magnified view of the low-intensity region of the scatter plot shown in Figure 3b in the main text.

S3. AFM image of ALD-Al_2O_3 on TFSA-treated 1L MoS_2

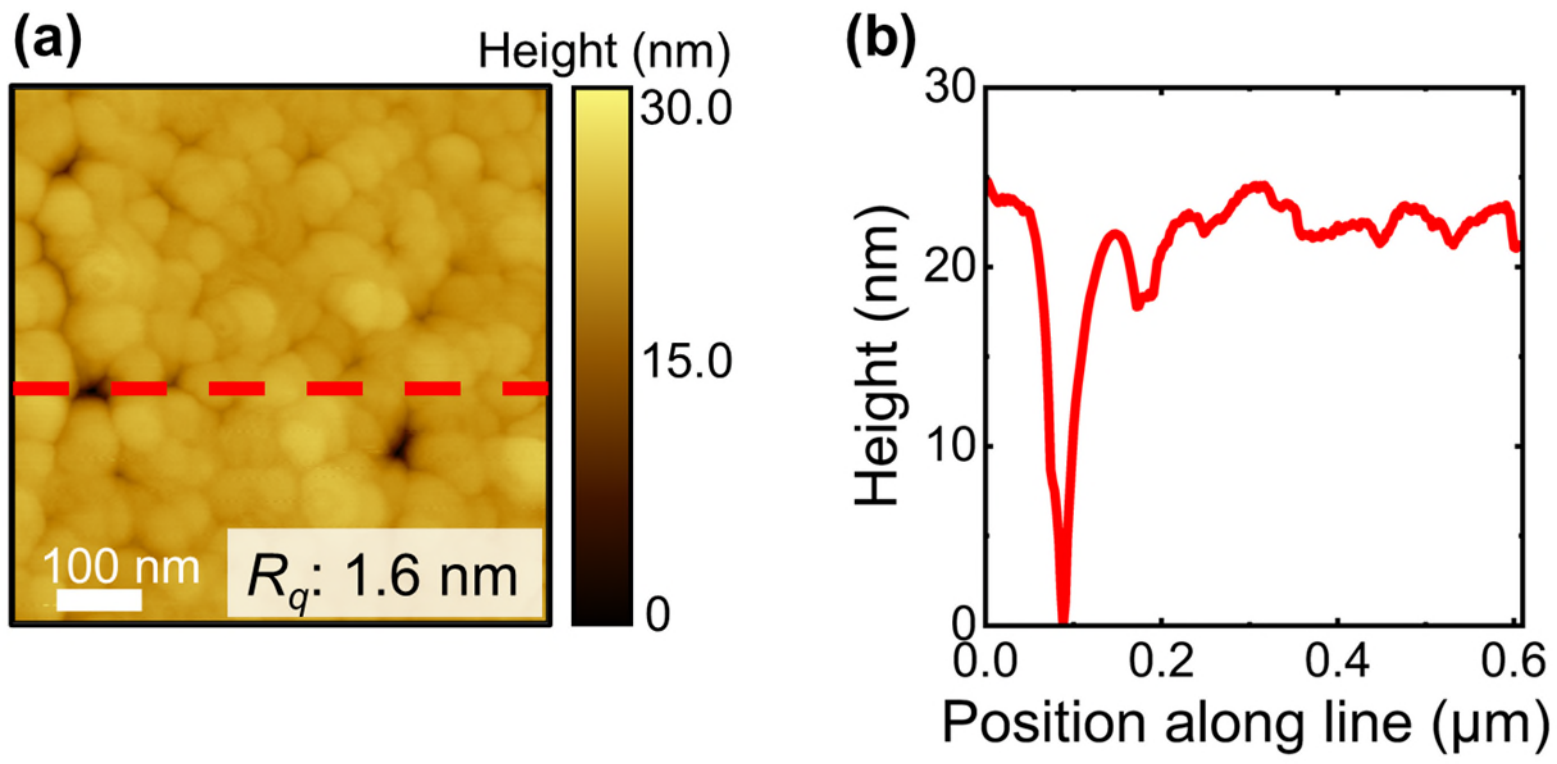

Figure S3: (a) High-magnification AFM image and (b) corresponding line profile of ALD-Al_2O_3 on TFSA-treated 1L MoS_2, deposited from 150 thermal ALD cycles. The line profile was traced along the red dashed line in (a).

S4. Deconvolution of single-spot PL spectra for ALD-Al_2O_3 on TFSA-treated 1L MoS_2

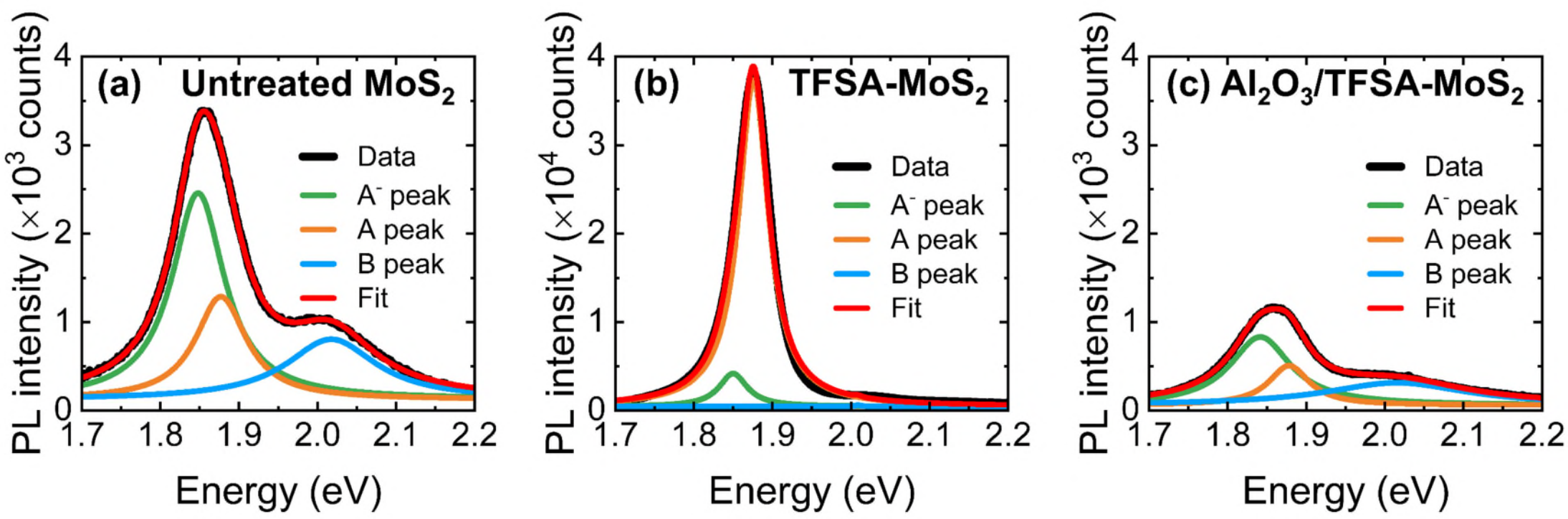

Figure S4: Multipeak Lorentzian fitting of the PL spectra obtained from 1L MoS_2 before and after TFSA treatment, and after subsequent thermal ALD of Al_2O_3. The cumulative fit, and contributions of the A^-, A, and B peaks are given in red, green, orange, and blue respectively. The differing intensity scales should be noted.

S5. PL mapping data for ALD control processes on TFSA-treated 1L MoS_2

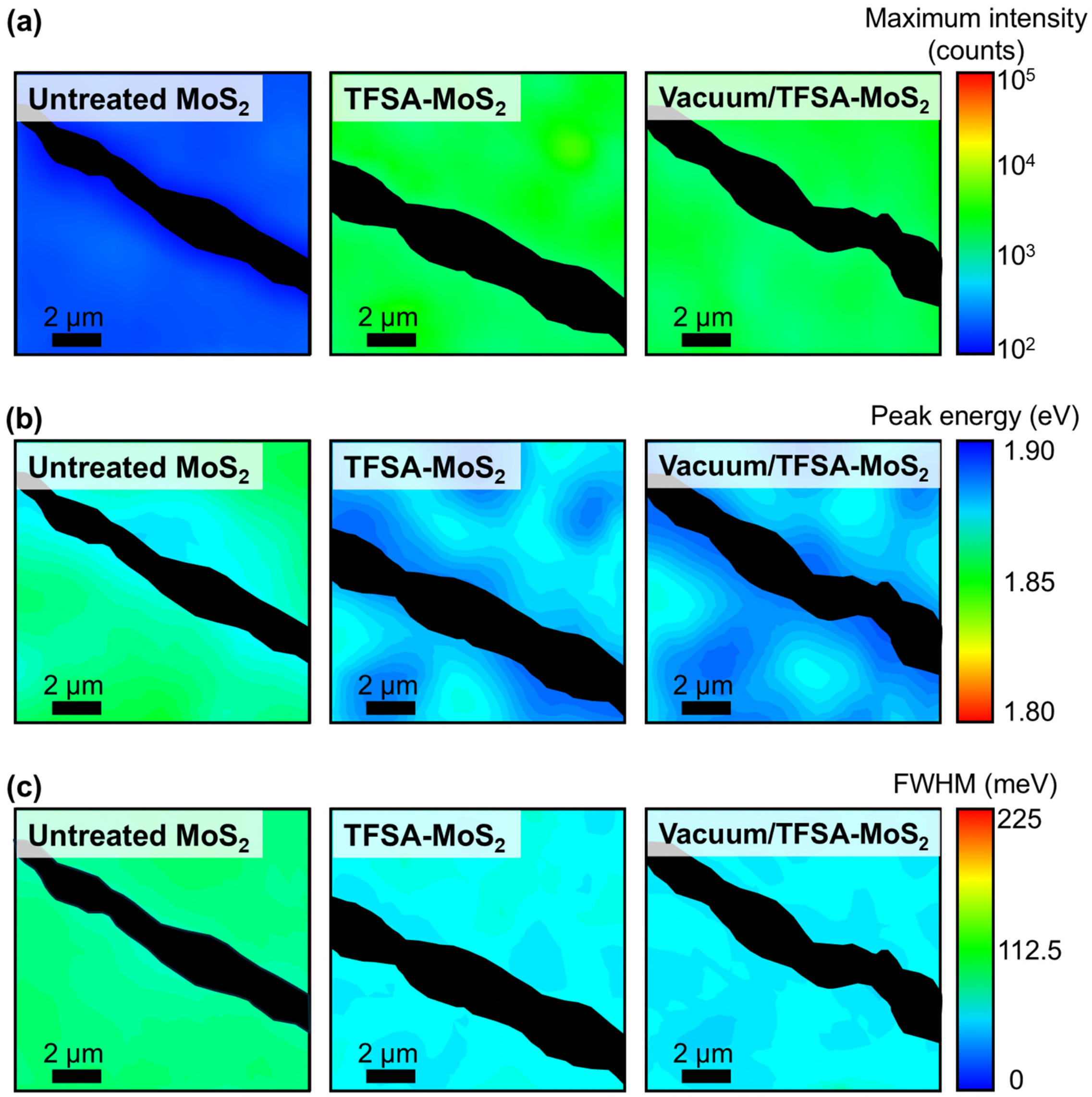

Figure S5: Maps of (a) maximum PL intensity, (b) peak PL energy, and (c) PL FWHM measured from 1L MoS_2 before and after TFSA treatment, and after subsequent vacuum exposure in an ALD system. No ALD growth process was performed. The black areas represent the SiO_2/Si substrate where no MoS_2 PL signal is detected.

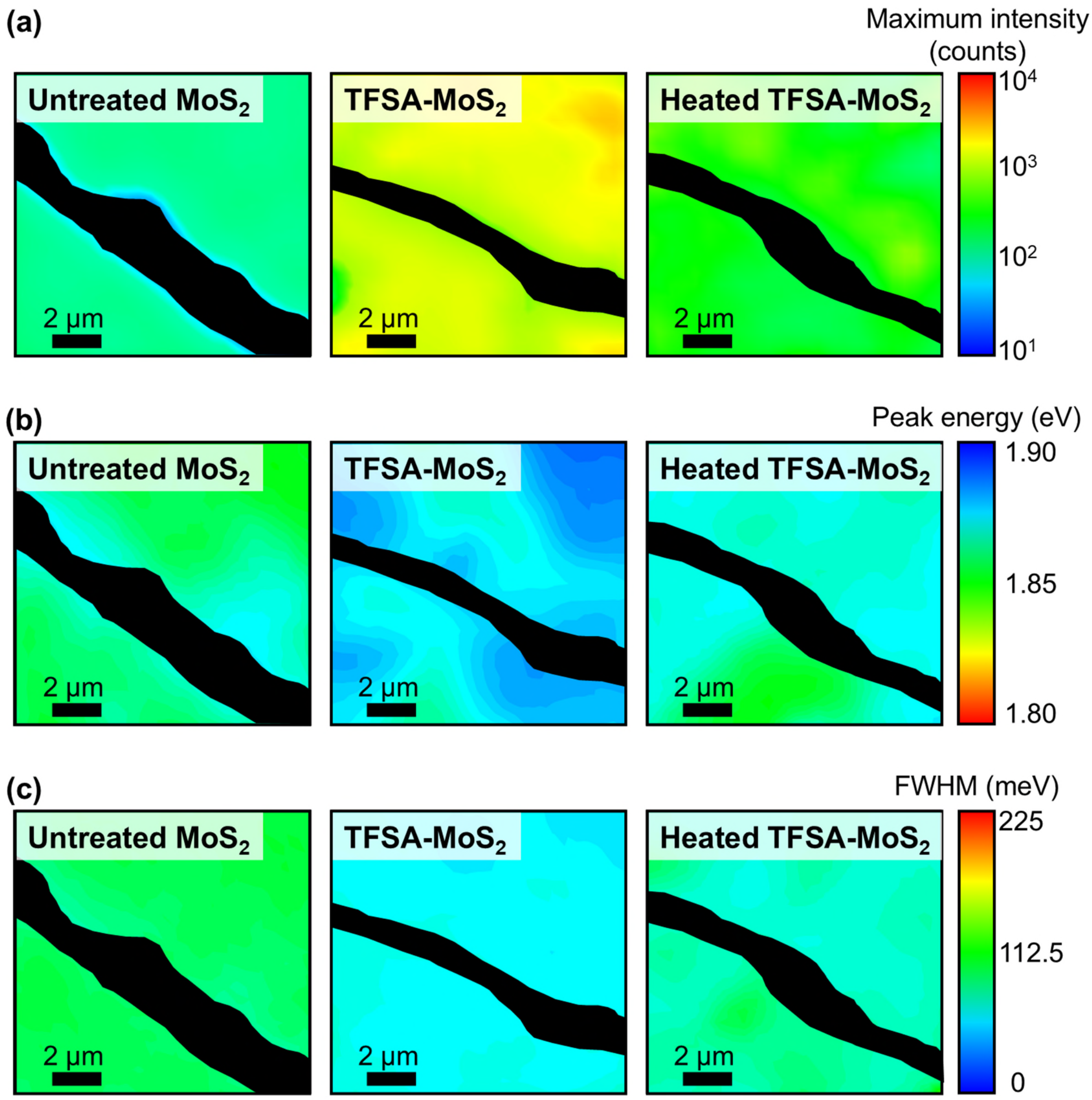

Figure S6: Maps of (a) maximum PL intensity, (b) peak PL energy, and (c) PL FWHM measured from 1L MoS_2 before and after TFSA treatment, and after subsequent heating under vacuum in an ALD system. No ALD growth process was performed. The black areas represent the SiO_2/Si substrate where no MoS_2 PL signal is detected.

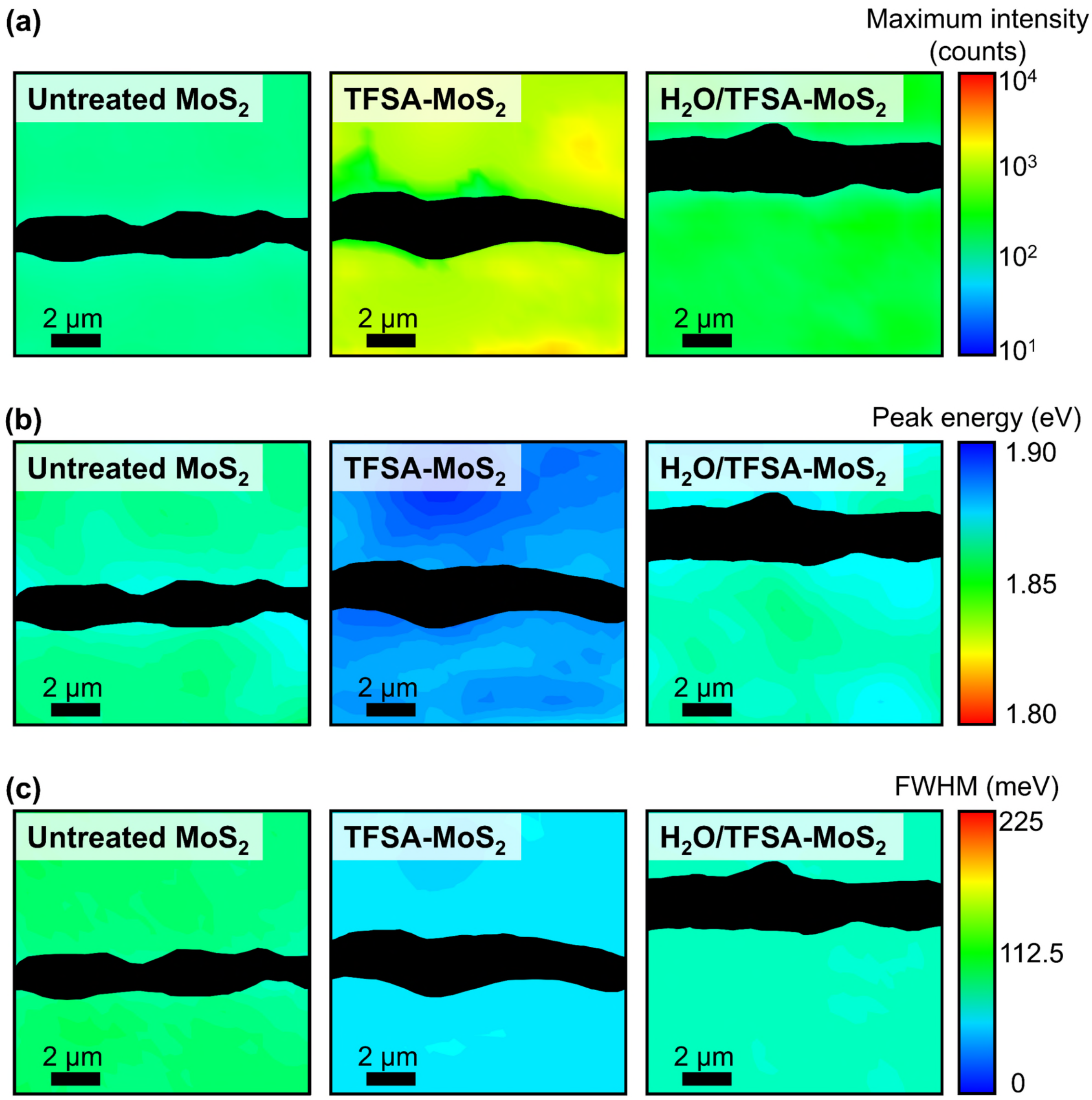

Figure S7: Maps of (a) maximum PL intensity, (b) peak PL energy, and (c) PL FWHM measured from 1L MoS_2 before and after TFSA treatment, and after subsequent pulsed exposure to H_2O at 150 °C in an ALD system. No TMA precursor pulses were included in this process, so no ALD growth was performed. The black areas represent the SiO_2/Si substrate where no MoS_2 PL signal is detected.

S6. Magnified PL scatter plots for ALD control processes on TFSA-treated 1L MoS_2

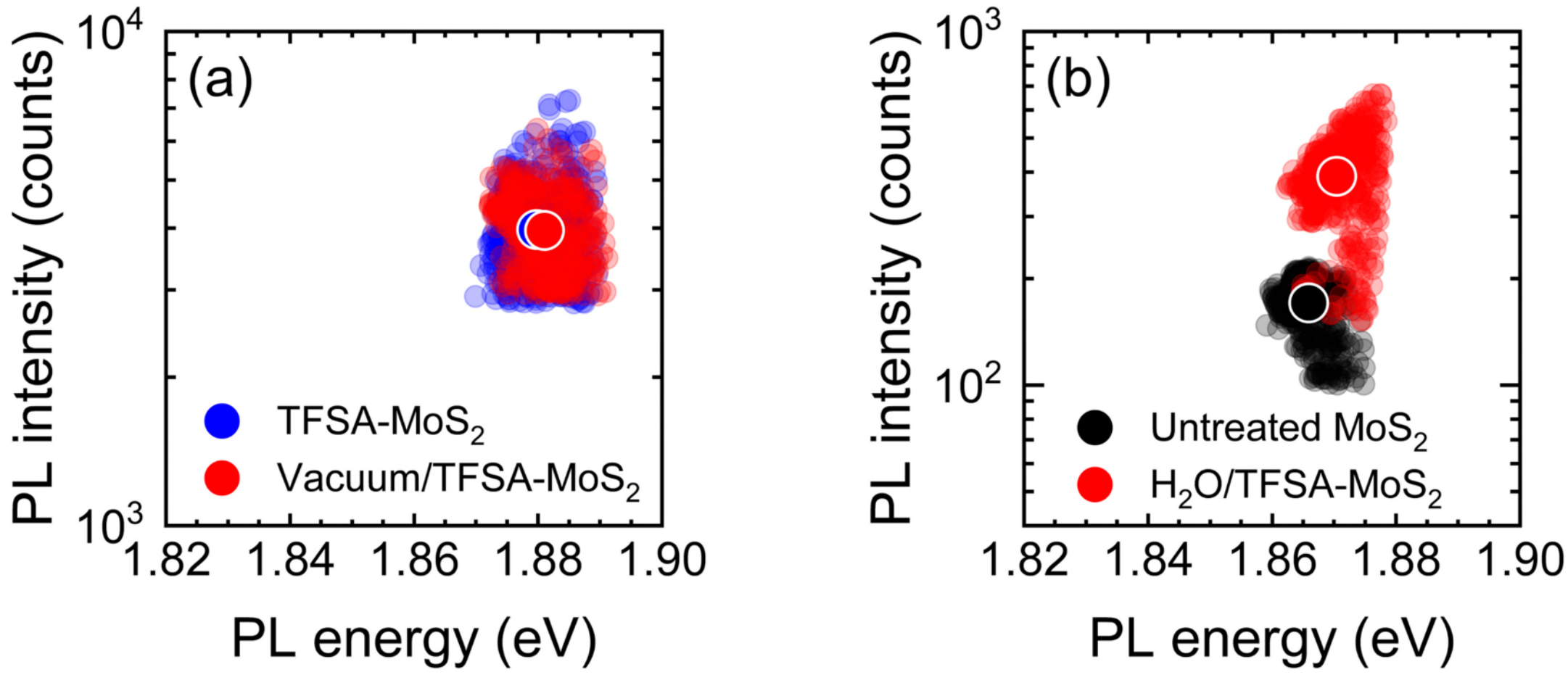

Figure S8: Magnified views of selected regions of the scatter plots (bottom) shown in Figures 4a and b in the main text.

S7. AFM images of TFSA-treated Al_2O_3/1L MoS_2

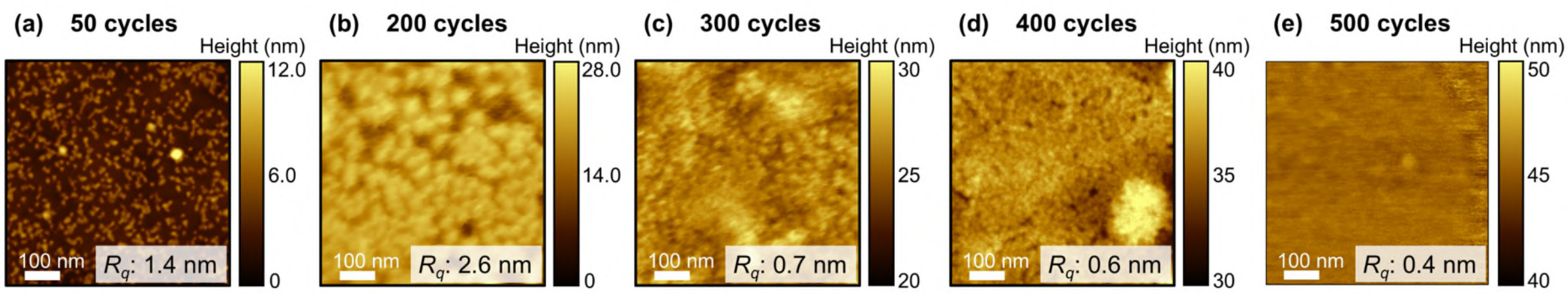

Figure S9: High-magnification AFM images of the TFSA-treated thermal ALD-Al_2O_3 films grown on 1L MoS_2 at cycle numbers (a) 50, (b) 200, (c) 300, (d) 400, and (e) 500. The root mean square (RMS) surface roughness, R_q, is indicated in each image.

S8. PL mapping data for TFSA treatment of Al_2O_3-encapsulated 1L MoS_2

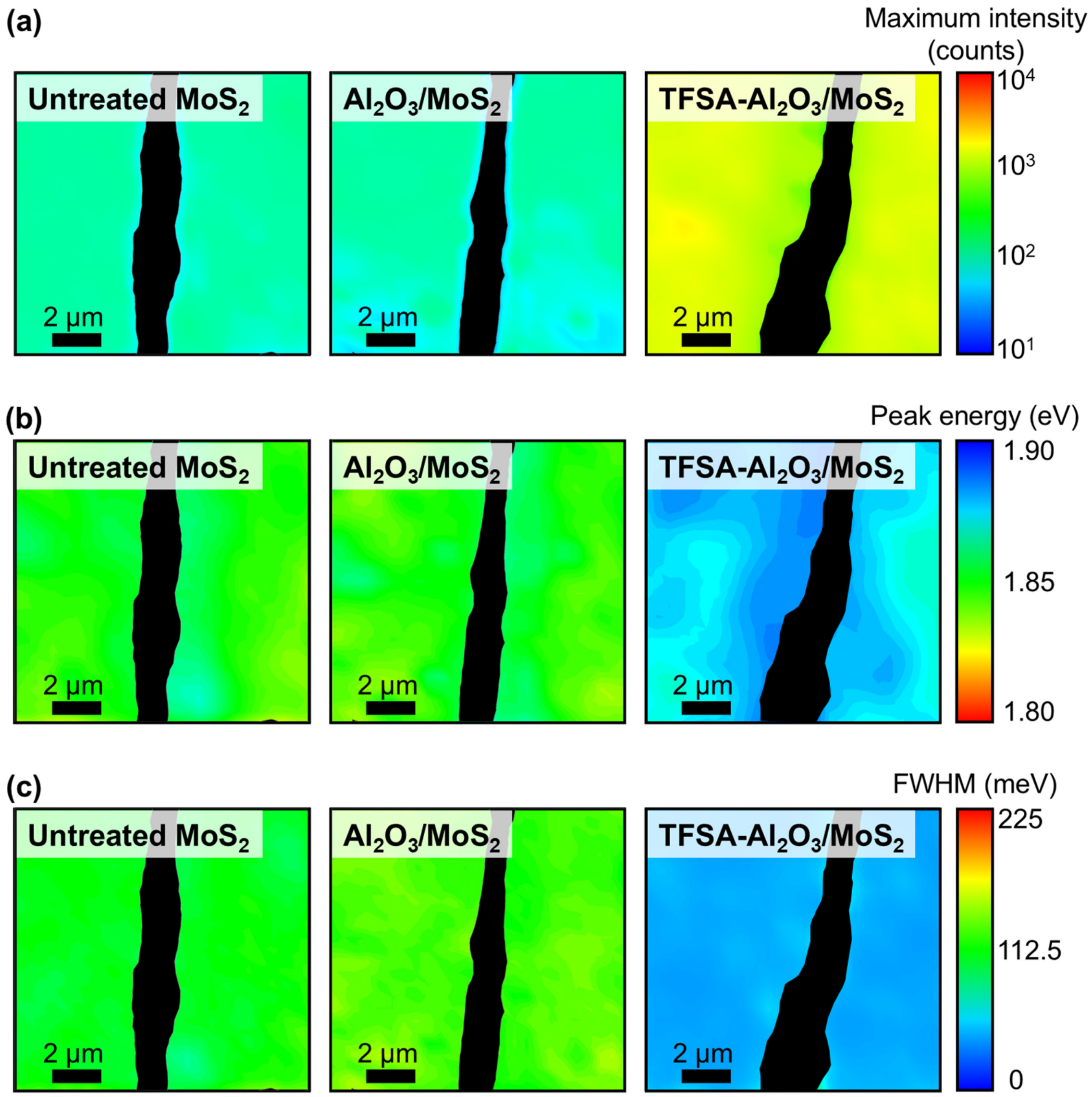

Figure S10: Maps of (a) maximum PL intensity, (b) peak PL energy, and (c) PL FWHM measured from 1L MoS_2 before and after 50 ALD cycles of thermal Al_2O_3, and after subsequent TFSA treatment. The black areas represent the SiO_2/Si substrate where no MoS_2 PL signal is detected.

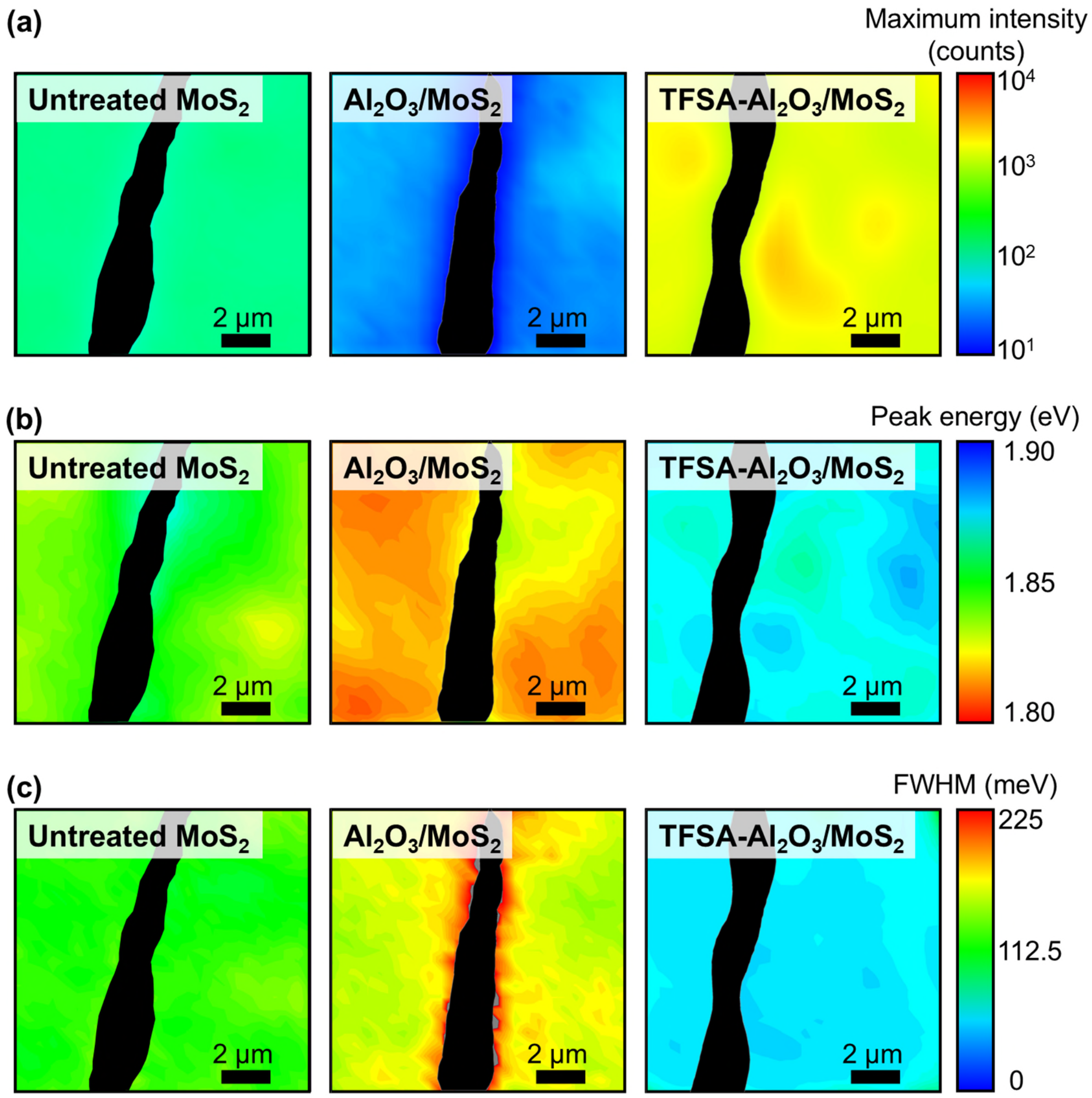

Figure S11: Maps of (a) maximum PL intensity, (b) peak PL energy, and (c) PL FWHM measured from 1L MoS_2 before and after 200 ALD cycles of thermal Al_2O_3, and after subsequent TFSA treatment. The black areas represent the SiO_2/Si substrate where no MoS_2 PL signal is detected.

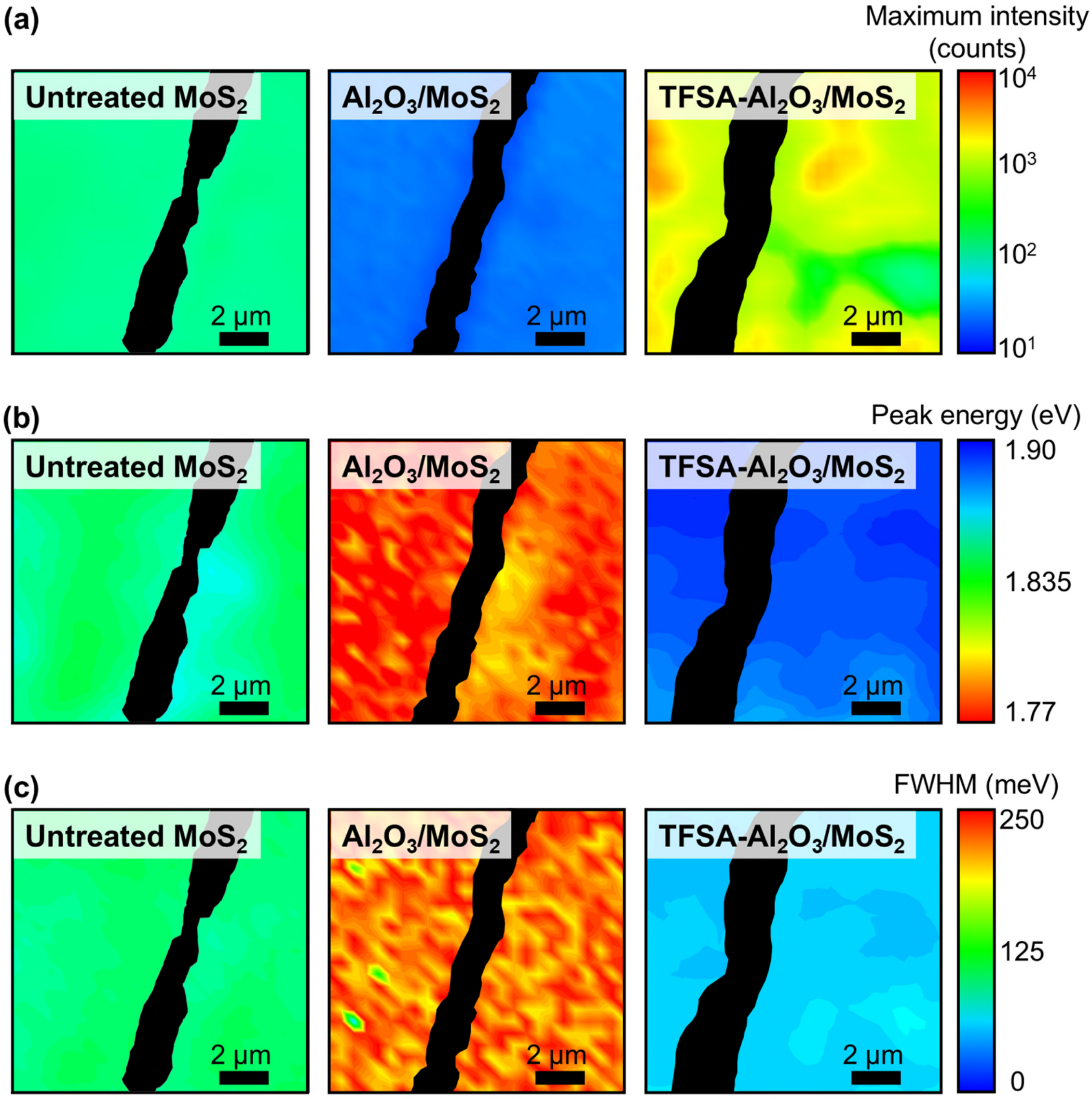

Figure S12: Maps of (a) maximum PL intensity, (b) peak PL energy, and (c) PL FWHM measured from 1L MoS_2 before and after 300 ALD cycles of thermal Al_2O_3, and after subsequent TFSA treatment. The black areas represent the SiO_2/Si substrate where no MoS_2 PL signal is detected.

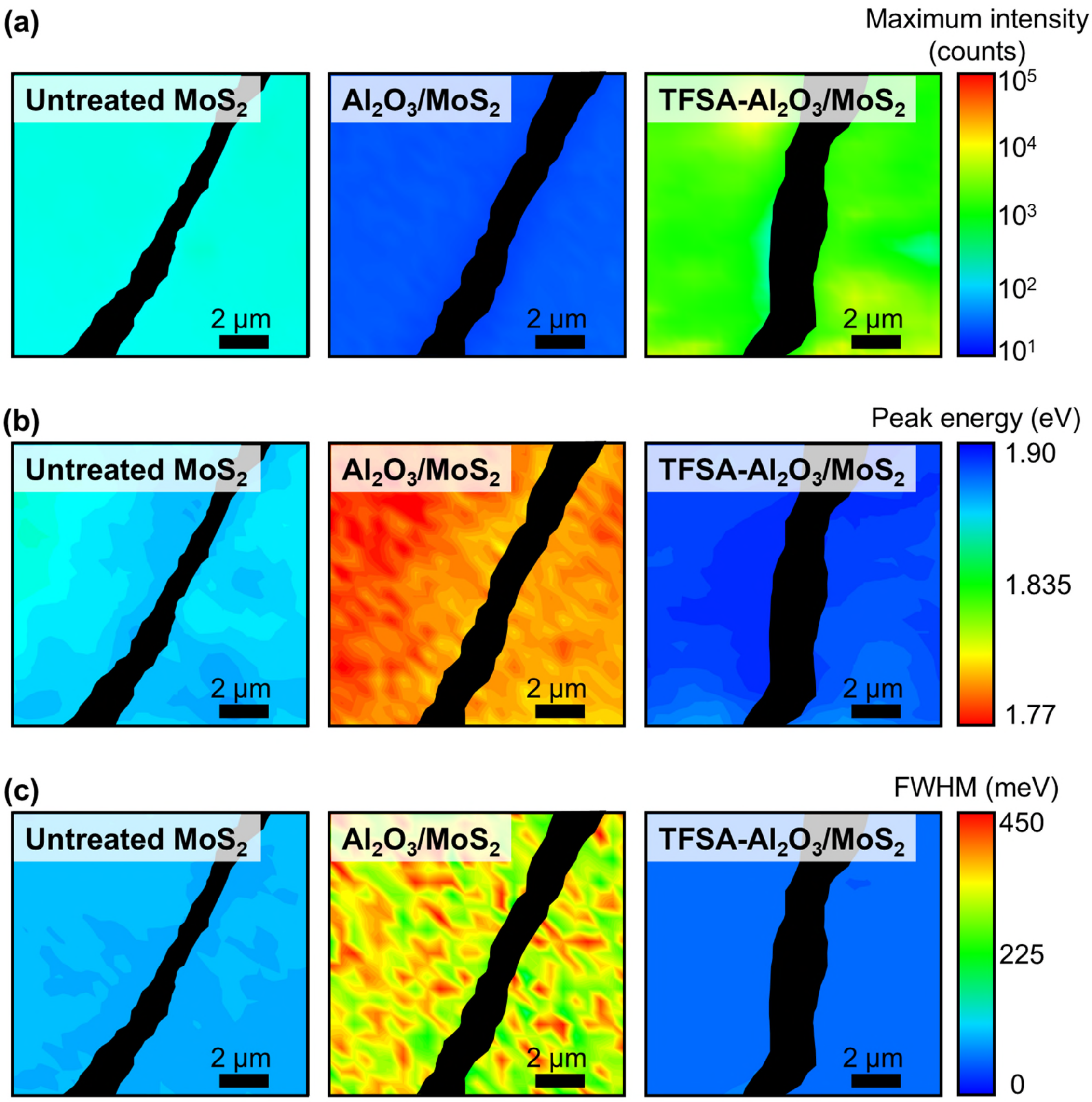

Figure S13: Maps of (a) maximum PL intensity, (b) peak PL energy, and (c) PL FWHM measured from 1L MoS_2 before and after 400 ALD cycles of thermal Al_2O_3, and after subsequent TFSA treatment. The black areas represent the SiO_2/Si substrate where no MoS_2 PL signal is detected.

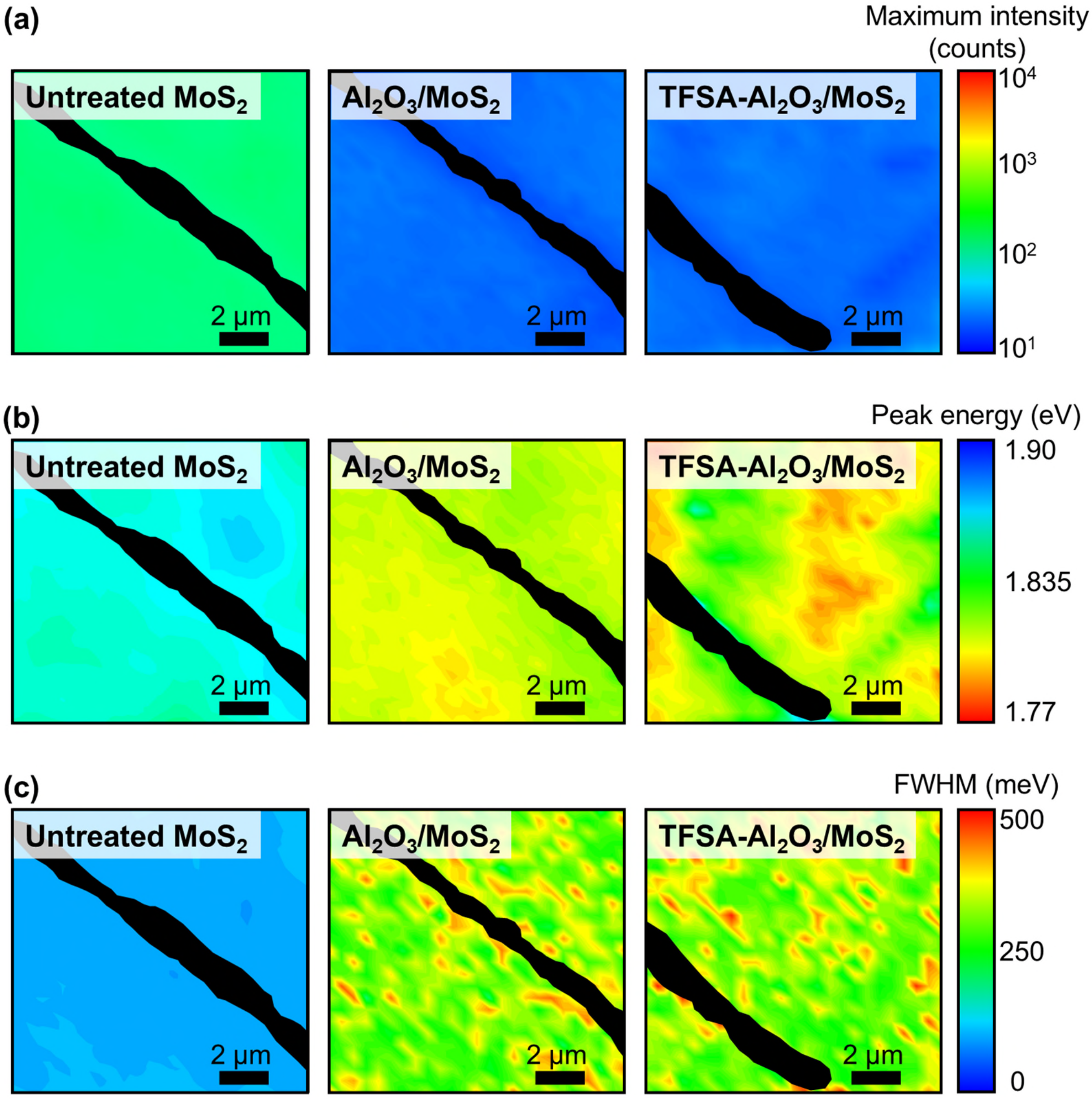

Figure S14: Maps of (a) maximum PL intensity, (b) peak PL energy, and (c) PL FWHM measured from 1L MoS_2 before and after 500 ALD cycles of thermal Al_2O_3, and after subsequent TFSA treatment. The black areas represent the SiO_2/Si substrate where no MoS_2 PL signal is detected.

S9. Deconvolution of single-spot PL spectra for TFSA treated Al_2O_3/1L MoS_2

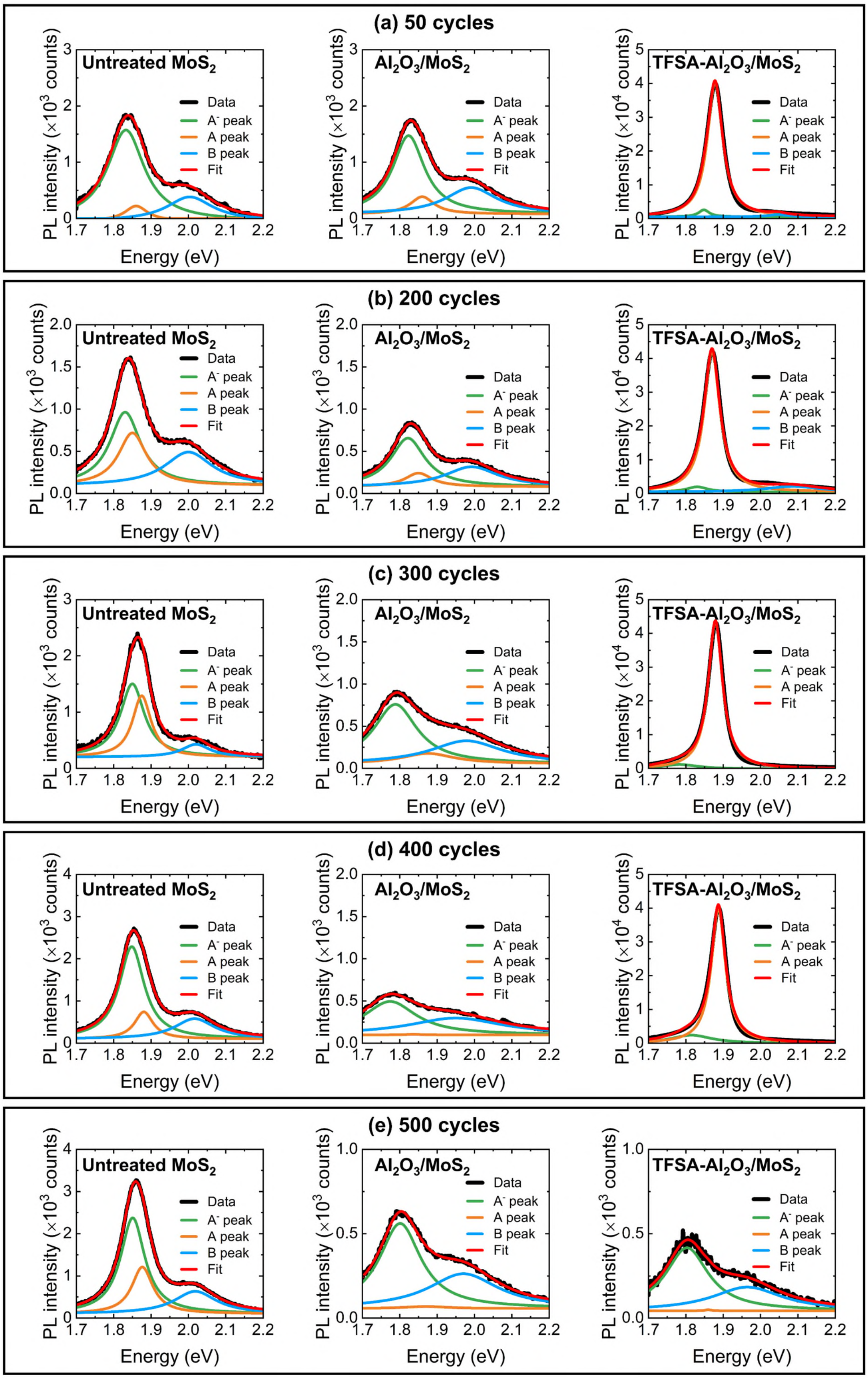

Figure S15: Multipeak Lorentzian fitting of the PL spectra obtained from 1L MoS_2 before and after thermal ALD of Al_2O_3, and after subsequent TFSA treatment, for ALD cycle numbers (a) 50, (b) 200, (c) 300, (d) 400, and (e) 500. The cumulative fit, and contributions of the A^-, A, and B peaks are given in red, green, orange, and blue respectively. The differing intensity scales should be noted.

S10. Raman mapping data for TFSA treatment of Al_2O_3-encapsulated 1L MoS_2

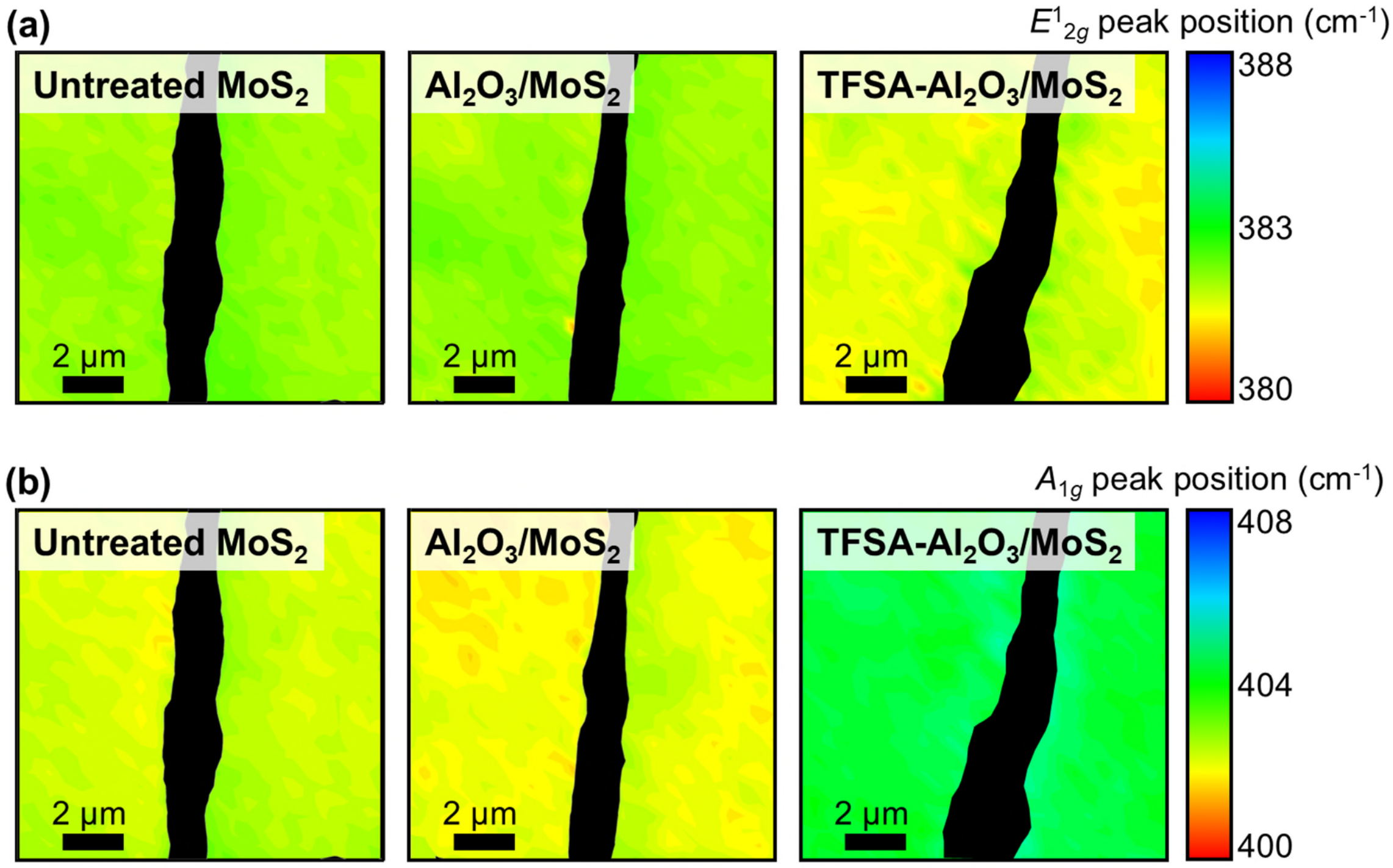

Figure S16: Maps of (a) E^1_{2g} and (b) A_{1g} Raman peak positions measured from 1L MoS_2 before and after 50 ALD cycles of thermal Al_2O_3, and after subsequent TFSA treatment. The black areas represent the SiO_2/Si substrate where no MoS_2 Raman signal is detected.

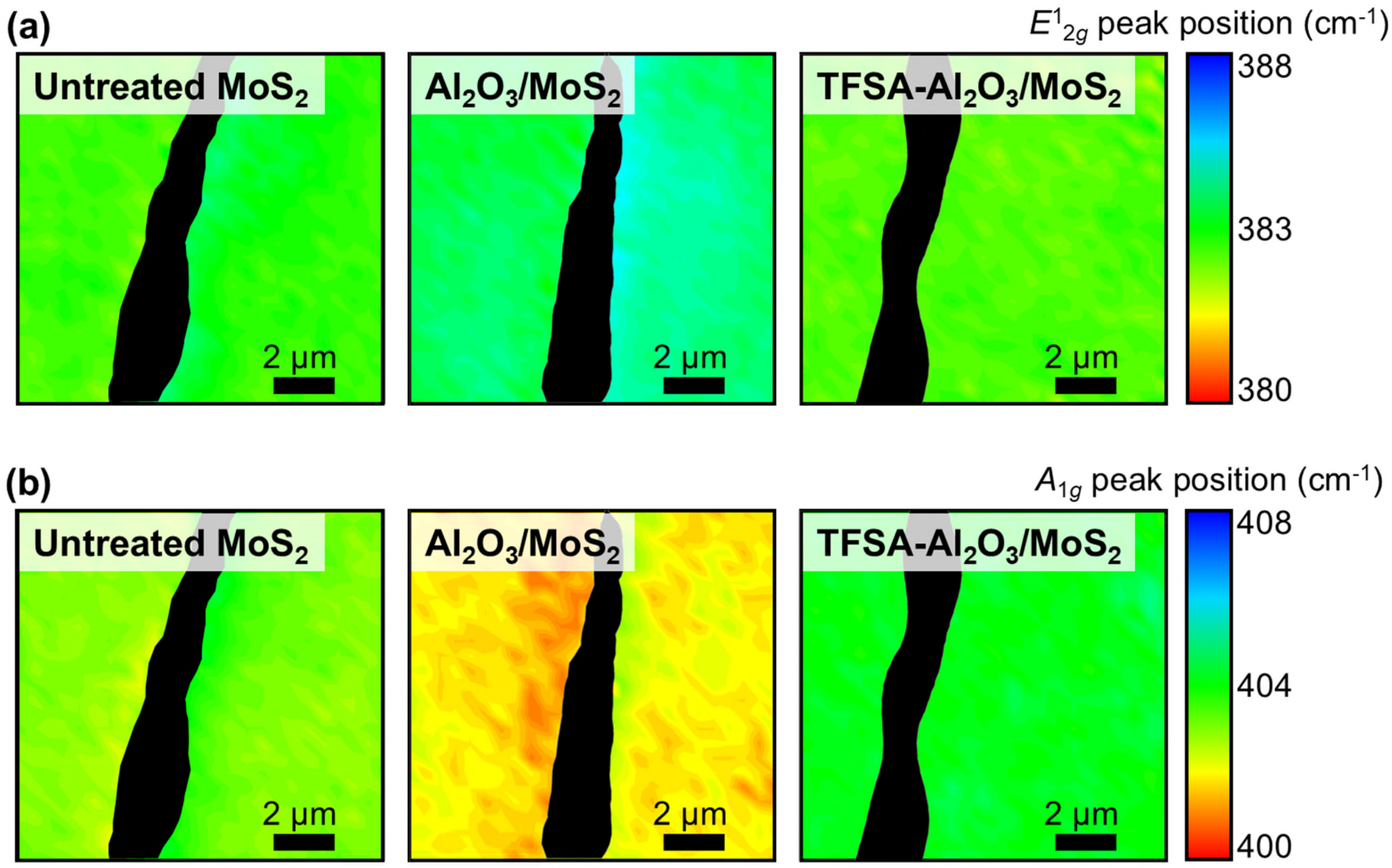

Figure S17: Maps of (a) E^1_{2g} and (b) A_{1g} Raman peak positions measured from 1L MoS_2 before and after 200 ALD cycles of thermal Al_2O_3, and after subsequent TFSA treatment. The black areas represent the SiO_2/Si substrate where no MoS_2 Raman signal is detected.

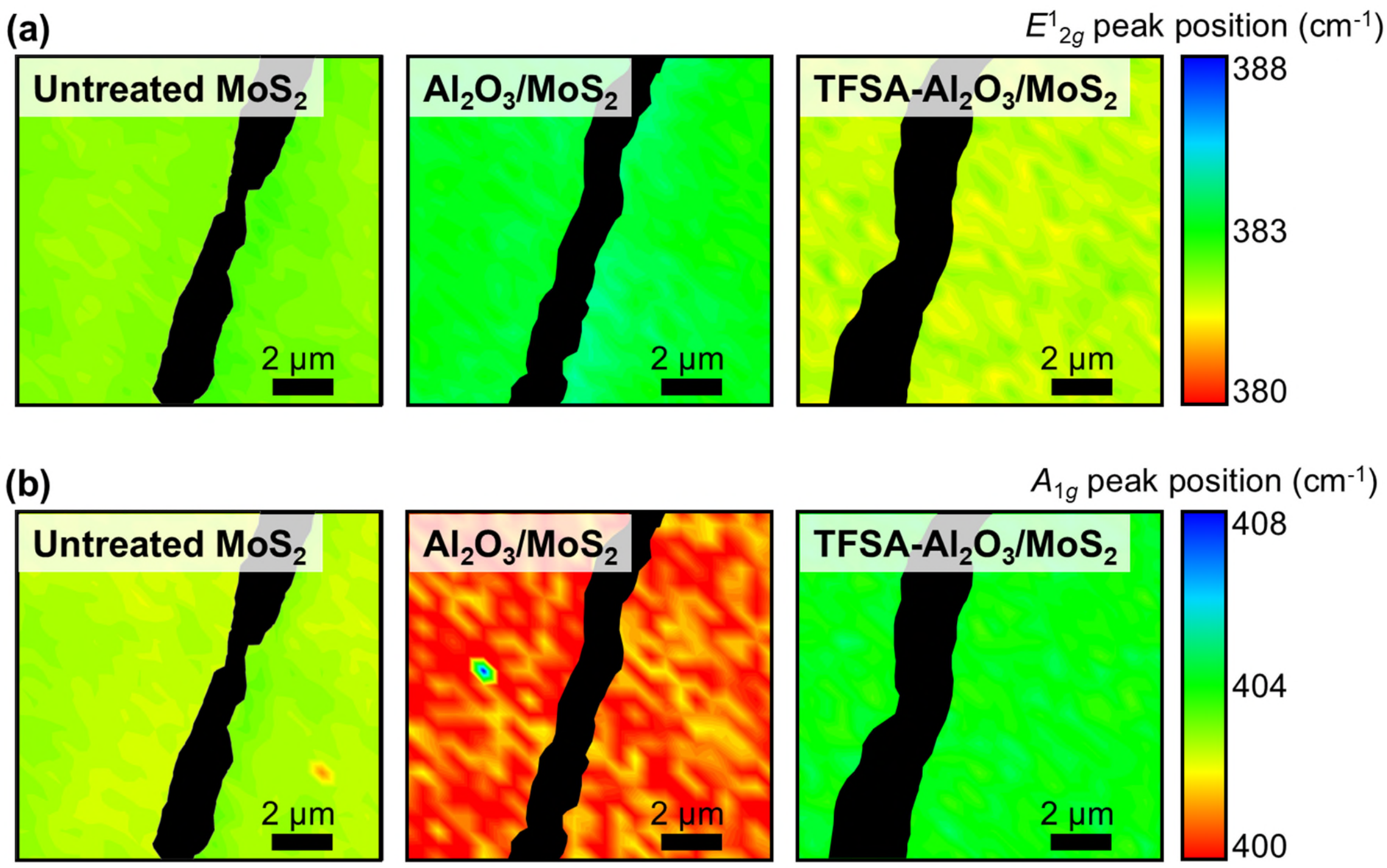

Figure S18: Maps of (a) E^1_{2g} and (b) A_{1g} Raman peak positions measured from 1L MoS_2 before and after 300 ALD cycles of thermal Al_2O_3, and after subsequent TFSA treatment. The black areas represent the SiO_2/Si substrate where no MoS_2 Raman signal is detected.

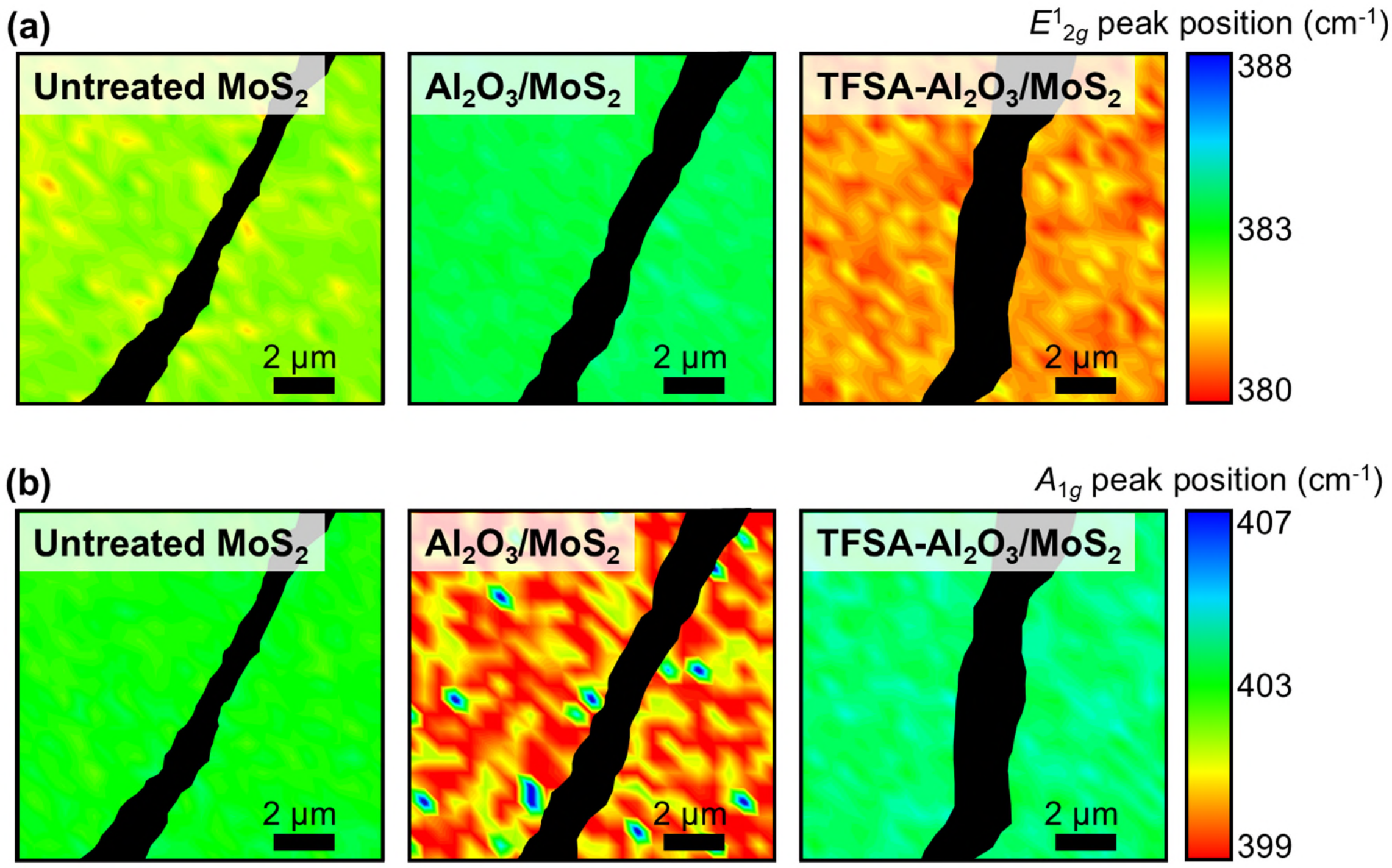

Figure S19: Maps of (a) E^{1}_{2g} and (b) A_{1g} Raman peak positions measured from 1L MoS_2 before and after 400 ALD cycles of thermal Al_2O_3, and after subsequent TFSA treatment. The black areas represent the SiO_2/Si substrate where no MoS_2 Raman signal is detected.

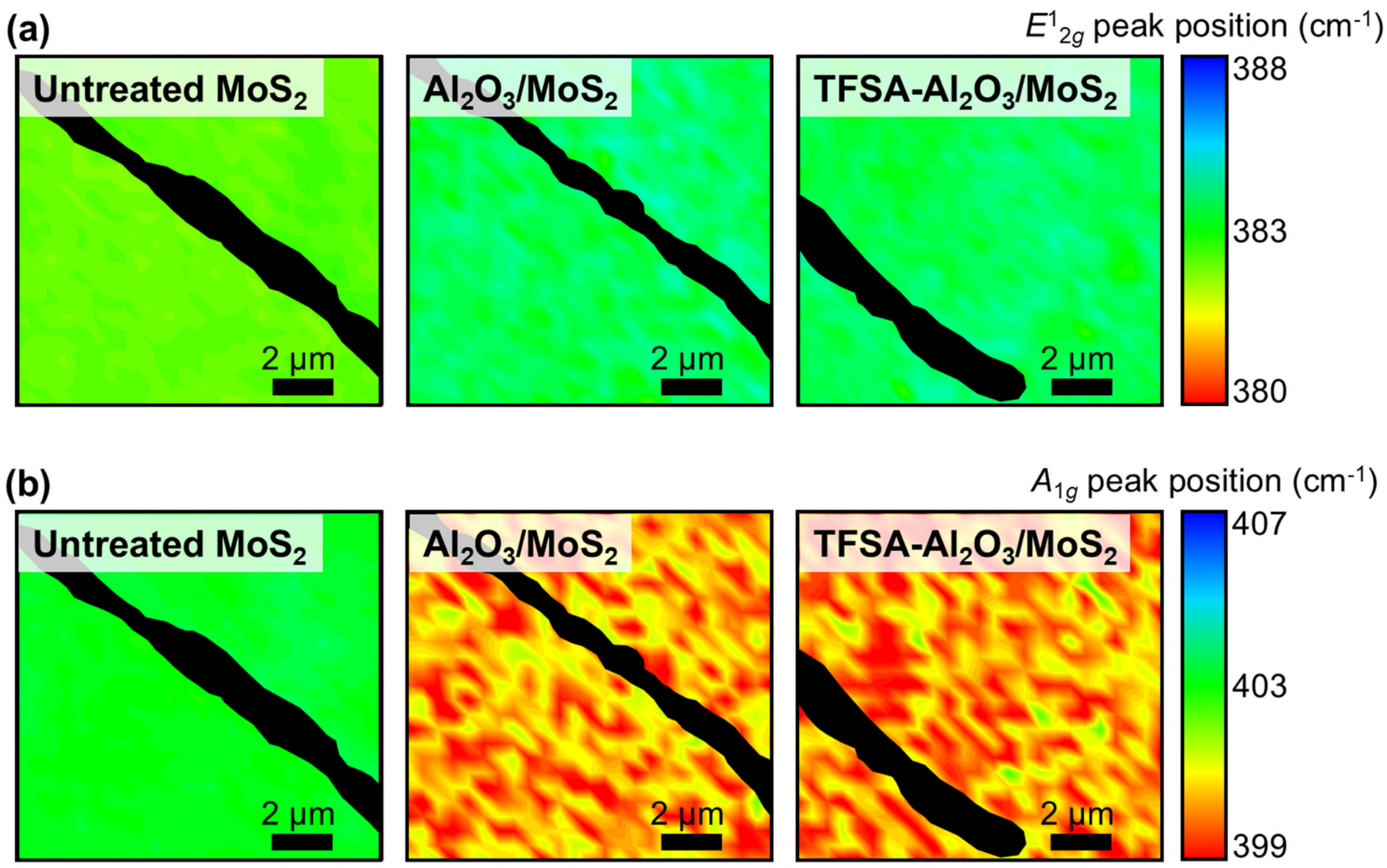

Figure S20: Maps of (a) E^{1}_{2g} and (b) A_{1g} Raman peak positions measured from 1L MoS_2 before and after 500 ALD cycles of thermal Al_2O_3, and after subsequent TFSA treatment. The black areas represent the SiO_2/Si substrate where no MoS_2 Raman signal is detected.

S11. Correlative analysis of strain and doping in 1L MoS_2

The following analysis has been previously performed by other authors for graphene [1-5] and more recently 1L MoS_2. [6-12] A freestanding MoS_2 monolayer is a good approximation of an ideal strain-free and undoped system. The characteristic $E^1{}_{2g}$ and A_{1g} Raman modes for suspended CVD-synthesised 1L MoS_2 appear at ~385 cm^{-1} and ~405 cm^{-1}, respectively, [8, 13] and are denoted $\omega^0_{E'}$ and $\omega^0_{A'}$. The positions of the $E^1{}_{2g}$ and A_{1g} peaks in the Raman spectrum of a strained and doped 1L MoS_2, $\omega_{E'}$ and $\omega_{A'}$, can be written in terms of the induced biaxial strain, ε, and electron density, n: [8]

$$\omega_{E'} = \omega^0_{E'} - 2\gamma_{E'}\omega^0_{E'}\varepsilon + k_{E'}n \quad \text{(S1)}$$

$$\omega_{A'} = \omega^0_{A'} - 2\gamma_{A'}\omega^0_{A'}\varepsilon + k_{A'}n \quad \text{(S2)}$$

where $\gamma_{E'}$ and $\gamma_{A'}$ are the Grüneisen parameters for the two characteristic Raman peaks in 1L MoS_2, and $k_{E'}$ and $k_{A'}$ are the corresponding shift rates of the vibrational modes as a function of carrier density, n. The relevant values used in this analysis are detailed in Table S1.

Table S1: Literature values for the Grüneisen parameters and Raman mode shift rates used in the correlative Raman analysis described in this work.

**Parameter**	**Value**	**Reference**
$\gamma_{E'}$	0.84	Rice *et al.* [14]
$\gamma_{A'}$	0.15	Rice *et al.* [14]
$k_{E'}$ ($\times 10^{-13}$ cm)	-0.33	Chakraborty *et al.* [15]
$k_{A'}$ ($\times 10^{-13}$ cm)	-2.22	Chakraborty *et al.* [15]

It follows from Equations S1 and S2, that any shifts in the positions of the characteristic $E^1{}_{2g}$ and A_{1g} Raman modes that result from a given perturbation can be expressed as: [6]

$$\Delta\omega_{E'} = -2\gamma_{E'}\omega^0_{E'}\varepsilon + k_{E'}n \quad \text{(S3)}$$

$$\Delta\omega_{A'} = -2\gamma_{A'}\omega^0_{A'}\varepsilon + k_{A'}n \quad \text{(S4)}$$

From this linear set of equations, we obtain relations for the strain and doping:

$$\varepsilon = \frac{k_{A'}\Delta\omega_{E'} - k_{E'}\Delta\omega_{A'}}{2\gamma_{A'}\omega_{A'}^{0}k_{E'} - 2\gamma_{E'}\omega_{E'}^{0}k_{A'}} \tag{S5}$$

$$n = \frac{\gamma_{A'}\omega_{A'}^{0}\Delta\omega_{E'} - \gamma_{E'}\omega_{E'}^{0}\Delta\omega_{A'}}{\gamma_{A'}\omega_{A'}^{0}k_{E'} - \gamma_{E'}\omega_{E'}^{0}k_{A'}} \tag{S6}$$

To determine the equation describing the strain isoline at zero doping, we set n = 0 and solve the system of Equations S1 and S2. Similarly, we can obtain the analogous equation for the strain-free doping line in the case of ε = 0.

$$\omega_{A'} = \omega_{A'}^{0} + \frac{\gamma_{A'}\omega_{A'}^{0}}{\gamma_{E'}\omega_{E'}^{0}}\left(\omega_{E'} - \omega_{E'}^{0}\right) \tag{S7}$$

$$\omega_{A'} = \omega_{A'}^{0} + \frac{k_{A'}}{k_{E'}}\left(\omega_{E'} - \omega_{E'}^{0}\right) \tag{S8}$$

Hence, we can generate a ε-n grid of strain and doping isolines, with respective gradients of $\frac{\gamma_{A'}\omega_{A'}^{0}}{\gamma_{E'}\omega_{E'}^{0}}$ = 0.19 and $\frac{k_{A'}}{k_{E'}}$ = 6.73, as shown in Figure S21. The dashed black lines represent the strain isolines and correspond to $\Delta\varepsilon$ = ±0.1 % variations in the strain. A $\Delta\varepsilon$ > 0 is indicative of tensile strain, whereas a negative $\Delta\varepsilon$ signifies compressive strain. Red dashes outline the doping isolines and indicate relative changes in the electron density of Δn = ±0.1 × 10^{13} cm^{-2}, where n-type doping is represented by Δn > 0 and p-type doping is implied by Δn < 0.

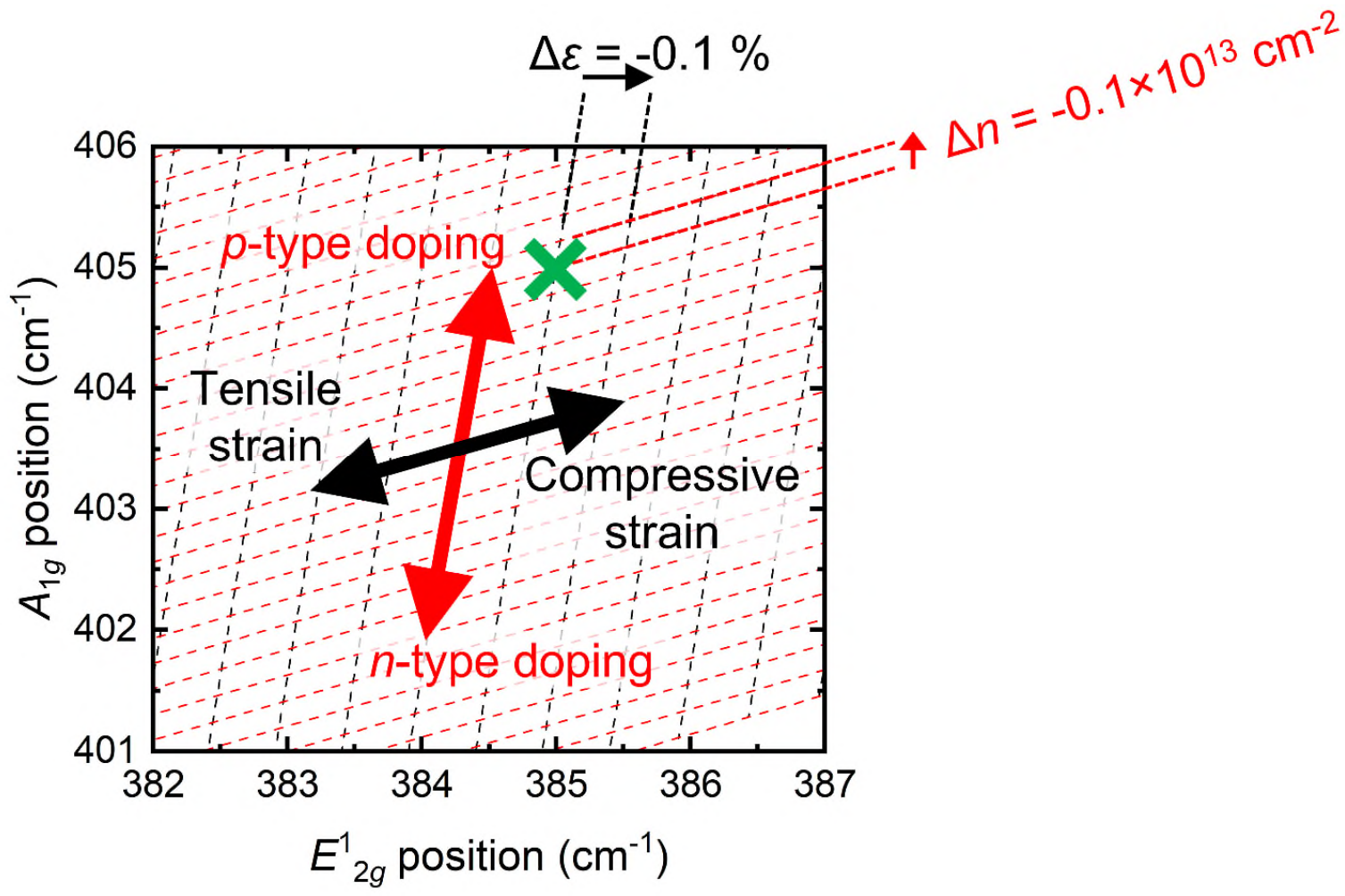

Figure S21: Representative correlative Raman plot of the A_{1g} Raman peak position as a function of the E^{1}_{2g} Raman peak position, with overlayed ε-n grid of isolines. The directions of the strain and doping effects indicated by the respective isolines are highlighted. The green cross indicates the intersection of the strain-free and undoped isolines at literature values of the Raman peak positions for a suspended CVD-synthesised 1L MoS_2 (E^{1}_{2g} ~385 cm^{-1} and A_{1g} ~405 cm^{-1}). Changes in strain and electron density relative to this point are indicated.

References

1. T. M. G. Mohiuddin, A. Lombardo, R. R. Nair, A. Bonetti, G. Savini, R. Jalil, N. Bonini, D. M. Basko, C. Galiotis, N. Marzari, K. S. Novoselov, A. K. Geim and A. C. Ferrari, *Phys. Rev. B*, 2009, **79**, 205433.
2. J. E. Lee, G. Ahn, J. Shim, Y. S. Lee and S. Ryu, *Nat. Commun.*, 2012, **3**, 1024.
3. C. Androulidakis, G. Tsoukleri, N. Koutroumanis, G. Gkikas, P. Pappas, J. Parthenios, K. Papagelis and C. Galiotis, *Carbon*, 2015, **81**, 322-328.
4. A. Armano, G. Buscarino, M. Cannas, F. M. Gelardi, F. Giannazzo, E. Schilirò and S. Agnello, *Carbon*, 2018, **127**, 270-279.
5. E. Schilirò, R. Lo Nigro, S. E. Panasci, F. M. Gelardi, S. Agnello, R. Yakimova, F. Roccaforte and F. Giannazzo, *Carbon*, 2020, **169**, 172-181.
6. A. Michail, N. Delikoukos, J. Parthenios, C. Galiotis and K. Papagelis, *Appl. Phys. Lett.*, 2016, **108**, 173102.
7. W. H. Chae, J. D. Cain, E. D. Hanson, A. A. Murthy and V. P. Dravid, *Appl. Phys. Lett.*, 2017, **111**, 143106.
8. S. E. Panasci, E. Schilirò, G. Greco, M. Cannas, F. M. Gelardi, S. Agnello, F. Roccaforte and F. Giannazzo, *ACS Appl. Mater. Interfaces*, 2021, **13**, 31248-31259.
9. E. Schilirò, S. E. Panasci, A. M. Mio, G. Nicotra, S. Agnello, B. Pecz, G. Z. Radnoczi, I. Deretzis, A. La Magna, F. Roccaforte, R. Lo Nigro and F. Giannazzo, *Appl. Surf. Sci.*, 2023, **630**, 157476.
10. E. Schilirò, R. L. Nigro, S. E. Panasci, S. Agnello, M. Cannas, F. M. Gelardi, F. Roccaforte and F. Giannazzo, *Adv. Mater. Interfaces*, 2021, **8**, 2101117.
11. B. F. M. Healy, S. L. Pain, J. Lloyd-Hughes, N. E. Grant and J. D. Murphy, *Adv. Mater. Interfaces*, 2024, **11**, 2400305.
12. B. F. M. Healy, S. L. Pain, N. E. Grant and J. D. Murphy, *Nanoscale*, 2025, **17**, 15436-15447.
13. D. Lloyd, X. Liu, J. W. Christopher, L. Cantley, A. Wadehra, B. L. Kim, B. B. Goldberg, A. K. Swan and J. S. Bunch, *Nano Lett.*, 2016, **16**, 5836-5841.
14. C. Rice, R. J. Young, R. Zan, U. Bangert, D. Wolverson, T. Georgiou, R. Jalil and K. S. Novoselov, *Phys. Rev. B*, 2013, **87**, 081307.
15. B. Chakraborty, A. Bera, D. V. S. Muthu, S. Bhowmick, U. V. Waghmare and A. K. Sood, *Phys. Rev. B*, 2012, **85**, 161403.
